# Magnetic Nanoparticles Mediated Thrombolysis–A Review

**DOI:** 10.1109/ojnano.2023.3273921

**Published:** 2023-05-08

**Authors:** BOHUA ZHANG, XIAONING JIANG

**Affiliations:** Department of Mechanical and Aerospace Engineering, North Carolina State University, Raleigh, NC 27695 USA

**Keywords:** Magnetic nanoparticles, magnetic microbubbles, thrombolysis, thrombolytic agents, sonothrombolysis, magneto-sonothrombolysis

## Abstract

Nanoparticles containing thrombolytic medicines have been developed for thrombolysis applications in response to the increasing demand for effective, targeted treatment of thrombosis disease. In recent years, there has been a great deal of interest in nanoparticles that can be navigated and driven by a magnetic field. However, there are few review publications concerning the application of magnetic nanoparticles in thrombolysis. In this study, we examine the current state of magnetic nanoparticles in the application of *in vitro* and *in vivo* thrombolysis under a static or dynamic magnetic field, as well as the combination of magnetic nanoparticles with an acoustic field for dual-mode thrombolysis. We also discuss four primary processes of magnetic nanoparticles mediated thrombolysis, including magnetic nanoparticle targeting, magnetic nanoparticle trapping, magnetic drug release, and magnetic rupture of blood clot fibrin networks. This review will offer unique insights for the future study and clinical development of magnetic nanoparticles mediated thrombolysis approaches.

## I. INTRODUCTION

Magnetic nanoparticles (MNPs) are nanoparticles that can be manipulated by magnetic fields. This manipulation can include attracting or repelling the MNPs using a magnetic field, aligning or reorienting their magnetic moments, or causing their magnetic moments to oscillate or rotate, depending on the type of magnetic field applied. There are four types of magnetic nanoparticles: ferrite nanoparticles, ferrites with a shell, metallic and metallic with a shell. Among them, ferrite nanoparticles, such as iron oxide nanoparticles, are the most widely used MNPs [1], [2], [3]. Besides, the surface of ferrite nanoparticles is usually coated with surfactants, silica, silicones, or phosphoric acid to increase the stability and dispersibility in complex fluids [4], [5]. For example, the ferrite nanoparticle clusters coated with a silica shell have higher chemical stability and narrow size distribution, beneficial for biomedical applications such as drug delivery and magnetic separation in bioengineering [6]. Moreover, single-domain iron oxide nanoparticles in the nanoscale may exhibit superparamagnetic behavior, which represents a promising tool for various biomedical applications such as hyperthermia, drug delivery, and magnetic resonance imaging (MRI) [7], [8], [9].

Many methods have been developed for the synthesis of MNPs, such as co-precipitation [10], thermal decomposition [11], and microemulsion [12]. Among them, the co-precipitation method has been widely used to synthesize different types of superparamagnetic iron oxide nanoparticles (SPIONs) with various compositions and coatings [13], [14], [15], [16], [17]. In addition, different organic coating materials such as fatty acids [18], polysaccharides [19], [20], [21], and polymer [22], [23], [24] have been used to increase the stability and biocompatibility of MNPs.

**FIGURE 1.**
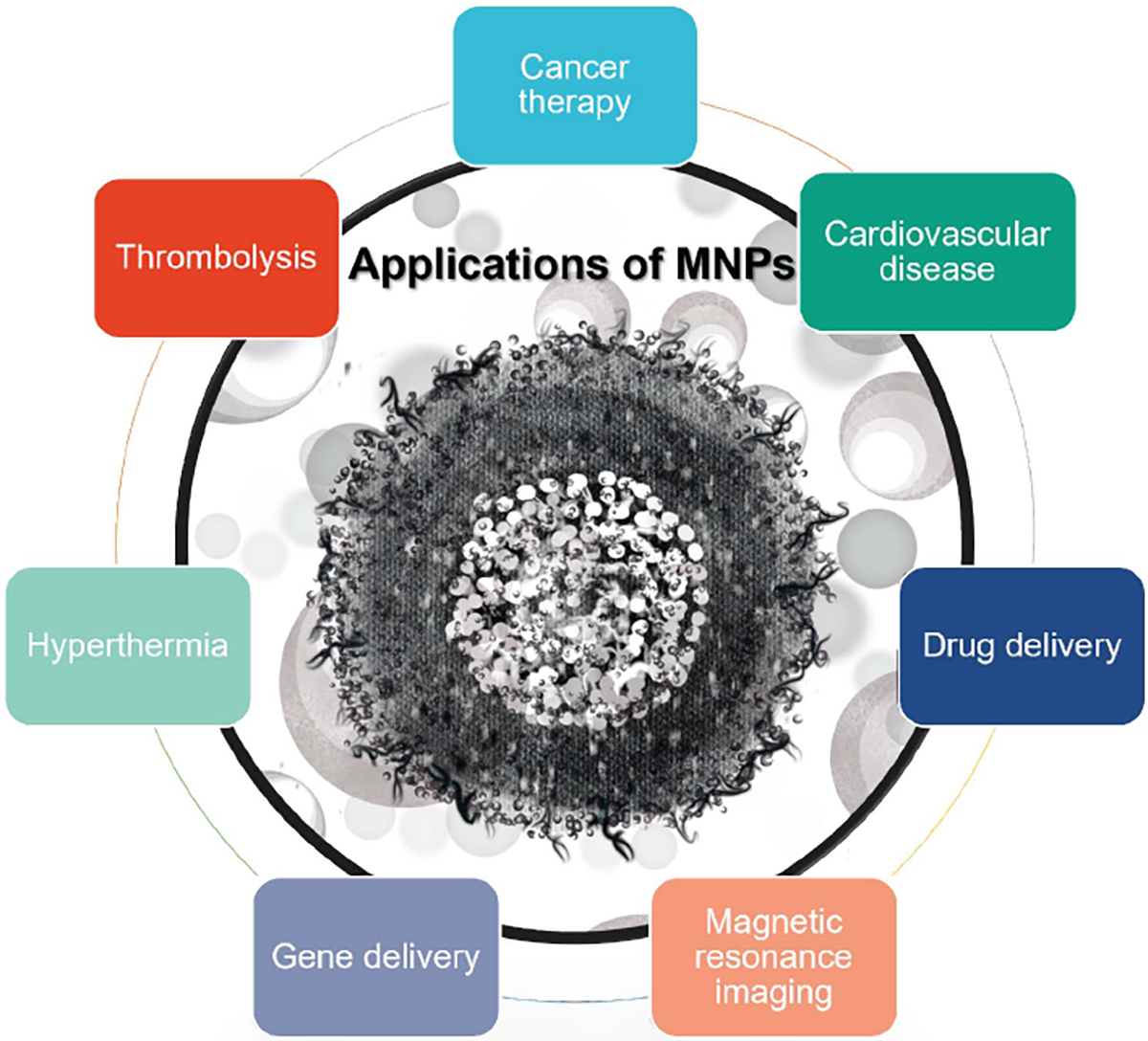
A mix of different applications of magnetic nanoparticles (MNPs) in medicine, and the types of pathologies or medical conditions they are used for.

As shown in Fig. 1, MNPs have been widely used in different medical applications, such as cancer therapy [25], [26], [27], [28] and cardiovascular disease [29], [30]. On one hand, MNPs can be used for diagnosis, such as the detection and characterization of atherosclerotic plaques [31], [32], myocardial infarction [33], and abdominal aortic aneurysms (AAAs) [34]. MNPs can be functionalized with specific molecules or ligands that can target these pathologies, allowing for more precise and sensitive detection [35]. On the other hand, MNPs can also be used for various biomedical therapies such as drug delivery [36], [37], [38], cardiac tissue engineering [39], gene delivery [40], photodynamic therapy [41], [42], and photothermal therapy [43].

Thrombosis is a medical condition characterized by the formation of blood clots inside blood vessels, which can obstruct blood flow and lead to various complications such as heart attack, stroke, pulmonary embolism, and deep vein thrombosis [44], [45]. The formation of blood clots is a natural process that helps to prevent bleeding in response to injury, but when it occurs in an abnormal or excessive manner, it can cause serious health problems [46], [47].

Thrombolysis is a medical treatment that aims to dissolve blood clots and restore blood flow to the affected tissues. It involves the administration of drugs called thrombolytics or fibrinolytics, which work by breaking down the proteins that hold blood clots together. Thrombolysis is commonly used to treat conditions such as acute ischemic stroke [48], myocardial infarction [49], and pulmonary embolism, where prompt restoration of blood flow is crucial to prevent tissue damage or organ failure. In addition to drug therapy, other methods of thrombolysis include mechanical thrombectomy [50], which involves the use of catheters or other devices to physically remove blood clots, and ultrasound-assisted thrombolysis, which uses high-frequency sound waves to enhance the activity of thrombolytic drugs.

Thrombosis and thrombolysis are important areas of research and clinical practice, as they play a critical role in the prevention and treatment of various cardiovascular and cerebrovascular diseases. Ongoing research is focused on developing more effective and targeted approaches to thrombolysis, including the use of magnetic nanoparticles for targeted drug delivery [51].

Magnetic nanoparticles have shown great promise for thrombolysis. First, magnetic nanoparticles can be designed to specifically target blood clots, allowing for a more targeted and efficient delivery of the therapeutic agent [52]. This targeted approach may reduce the risk of off-target effects and minimize damage to healthy tissue [53]. Second, Magnetic nanoparticles can be coated with thrombolytic agents, such as tissue plasminogen activator (tPA), which are known to dissolve blood clots [54]. The nanoparticles can be designed to release the thrombolytic agent in response to a magnetic field, which can enhance the rate and efficiency of thrombolysis [55]. Third, traditional thrombolytic agents can increase the risk of bleeding, which is a serious side effect. However, the use of magnetic nanoparticles may reduce this risk by allowing for more targeted and controlled delivery of the therapeutic agent [56]. Last, magnetic nanoparticles can be administered using a non-invasive approach, such as intravenous injection, which is less invasive than traditional approaches to thrombolysis, such as catheter-based procedures [57].

In this article, the mechanisms of MNPs-enhanced thrombolysis will be summarized first and then the current state of MNPs in thrombolysis application will be reviewed. Finally, the future perspectives of MNPs in thrombolysis will be discussed.

## II. MECHANISM OF MNPS ENHANCED THROMBOLYSIS

**FIGURE 2.**
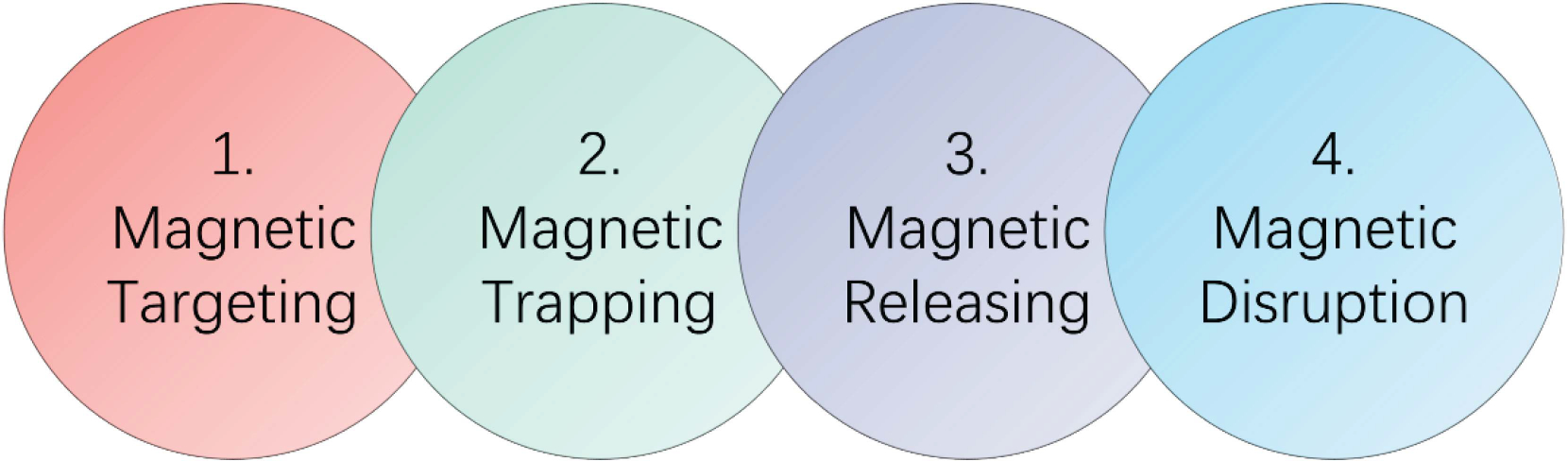
Mechanism of magnetic nanoparticles enhanced thrombolysis. The process includes magnetic targeting of thrombolytic drugs, magnetic trapping of drug loaded magnetic nanoparticles, magnetic releasing for the drugs and magnetic disruption of the fibrin networks of clot.

The fundamental mechanism of MNPs enhanced thrombolysis could be primarily associated with the following four factors including magnetic targeting, magnetic trapping, magnetic releasing, and magnetic disruption, as shown in Fig. 2. First, the MNPs were targeted to the local clot site under magnetic field manipulation. Second, the drug-loaded magnetic particles were trapped under the magnetic field against the blood flow, consequently increasing the local concentration of MNPs. Third, the drug-loaded magnetic particles could interact with clots under the dynamic magnetic field and release the thrombolytic drugs from the magnetic carrier. Finally, the released drugs could induce fibrinolysis, and the mechanical force from rotating magnetic particles under a dynamic magnetic field could further destroy the fibrin network of the blood clot and increase the thrombolytic efficacy.

**FIGURE 3.**
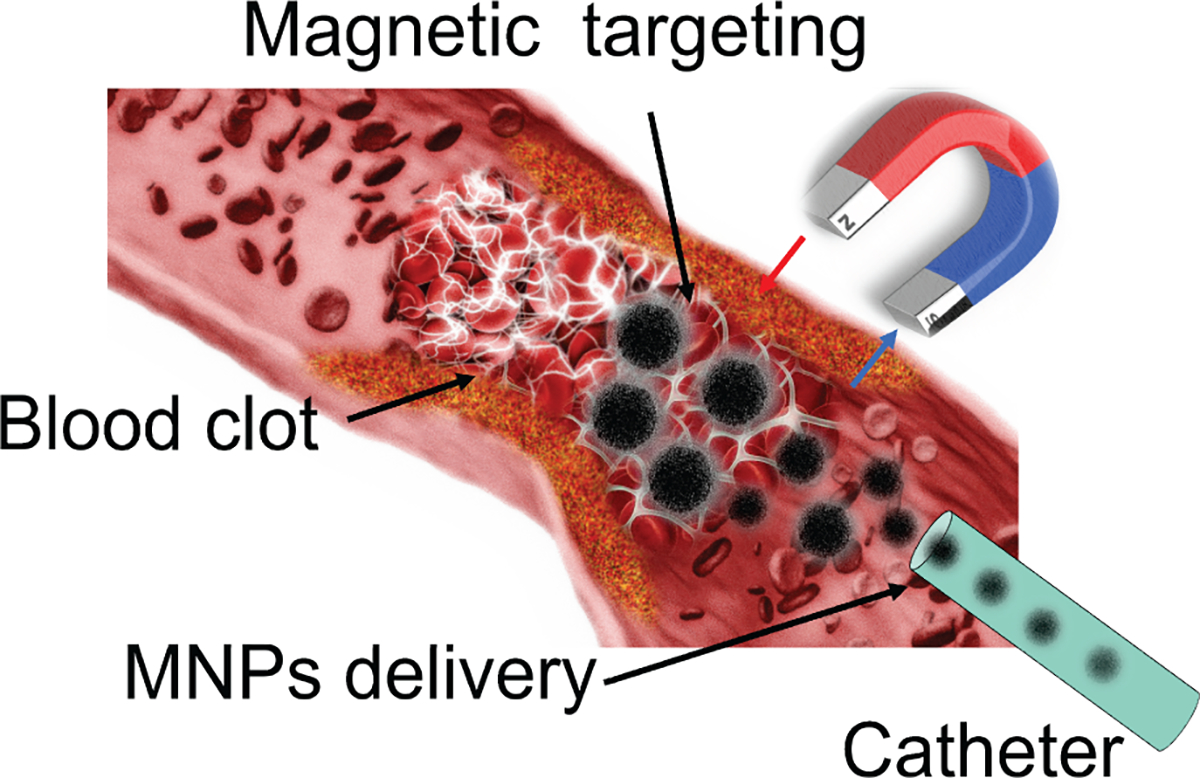
Illustration of magnetic targeting process: magnetic nanoparticles were delivered through a catheter and targeted at the clot site under magnetic force.

MNPs usually have a magnetite core with superparamagnetic properties and a polymer coating layer that offers better thrombolytic drug binding capability and a shield for increased stability in plasma. In other words, combining MNPs and thrombolytic drugs can provide drugs with the preferred magnetic property. As illustrated in Fig. 3, the MNPs carried with drugs can be delivered through a catheter and magnetically concentrated around the target region using a permanent external magnet or electromagnetic field. Moreover, the tPA-bonded MNPs can be chemically and colloidally stable so that the enzymatically active drugs can be delivered to a specific area in blood circulation and more effectively target fibrin clots. As a result, the MNPs can be further manipulated under the magnetic field, penetrate deeper fibrin matrices, and release tPA drugs within the clot to achieve a higher clot lysis rate.

**FIGURE 4.**
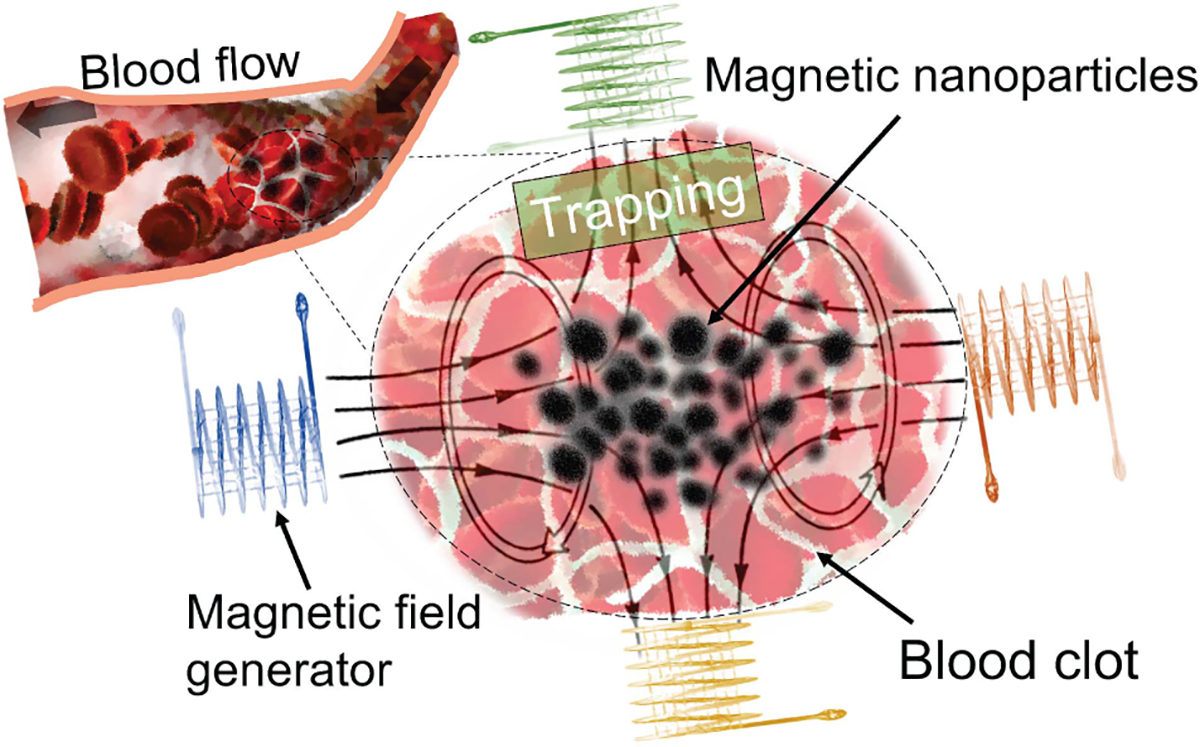
Illustration of the magnetic nanoparticle trapped on targeted clot site under magnetic field.

Since the MNPs are selectively delivered to the clot site by external magnetic force in a continuously circulating blood environment, the MNPs should be sufficiently magnetic for targeting with the external magnetic field gradients. For instance, as shown in Fig. 4, the MNPs can be trapped at the targeted clot region under magnetic fields against both venous and arterial flow. Moreover, the stability of tPA fibrinolytic drug in blood circulation has been dramatically enhanced by magnetic carriers, reducing the side effects and increasing the efficiency of thrombolytic treatment. Primarily, the MNPs can be targeted to the clot site and monitored under MRI to achieve detailed location information of the clot for detecting and examining the thrombolysis efficiency during the treatment.

The concentration of released thrombolytic agent should be high enough to induce the clot lysis. However, the primary concern of tPA-loaded magnetic carriers is the long-term stability since most of the recombinant tPA solutions are only stable for 24 hours at low temperatures. The short stability period of tPA solutions is a known challenge in the development of tPA-based therapies, including those involving magnetic carriers. Therefore, efforts are being made to improve the stability and shelf-life of tPA solutions in order to make them more viable for clinical use. One way to solve this problem is to encapsulate the thrombolytic agents inside the magnetic carriers with non-releasing behavior, and the tPA drugs can be protected and concentrated under a magnetic field through blood circulation. Similarly, the protein coupled MNPs may provide better bioavailability to reduce the total dosage. Furthermore, the effects of plasma inhibitors around the clot region can be countered by the high local concentrations of tPA on the surface of the MNPs. Therefore, the thrombolysis time can be extended, and the systemic fibrinolysis can be significantly reduced. However, the enzyme needs to penetrate the clot fibrin networks to achieve deeper fibrinolysis.

The MNPs would obtain dipole moments under an external magnetic field and have strong interactions among the magnetized dipolar nanoparticles, leading to clusters such as micro-chains or micro-rods particles. Besides, the shape of the micro-clusters is primarily dependent on the external magnetic field strength and frequency. In this case, the MNPs can be steered into the deep fibrin networks under the direction of the steepest magnetic field gradient. Moreover, the rotational or oscillating magnetic field can induce a magnetic torque for controlling the rotation speed of MNPs. For example, the low-velocity field produced by the rotating MNPs located far from the magnetic field source could only destroy the small fibrin structures from the narrow occlusion area. However, rotating MNPs towards the steepest gradient direction could have a stronger velocity field that helps break down larger clot structures more efficiently. The principle based on the fact that the strength of the magnetic field gradient decreases with distance from the magnetic field source, and thus the force applied to the MNP decreases with distance as well. As a result, MNPs located far from the magnetic field source would experience a weaker force and thus generate a lower velocity field. On the other hand, if the MNPs are rotated towards the steepest gradient direction, they would experience a stronger force and generate a stronger velocity field, which could help break down larger clot structures more efficiently. For example, the principle of using a non-uniform magnetic field to manipulate magnetic nanoparticles is based on the fact that the strength of the magnetic field gradient decreases with distance from the magnetic field source, and thus the force applied to the magnetic nanoparticles decreases with distance as well, which can affect the retention time of the nanoparticles in the blood flow and their ability to break down clots depending on the velocity of the blood flow [58].

The principle that the strength of the magnetic field gradient decreases with distance from the magnetic field source is a fundamental concept in the manipulation of micro- and nanostructures using magnetic fields, which has been discussed in another study [59]. The principle of the magnetic field gradient is also used in a magnetic manipulation system, which utilizes multiple soft-magnetic-core electromagnets to produce a gradient in magnetic field strength that decreases with distance from the source, allowing for precise control of micro- and nanostructures by generating a lower velocity field for structures located far from the magnetic field source [60]. In another study, paramagnetic beads are used as magnetic carriers for detection and manipulation of single molecules on a common platform, and the beads can be manipulated on-chip via currents running through specially designed line patterns based on the magnetic field gradient [61].

Moreover, the design of a magnetic manipulation system for medical devices requires sufficient magnetic fields and gradients to manipulate the target agent, and this work evaluates the forces needed to manipulate magnetic devices in bodily fluids and optimizes the magnetic systems comprised of multiple permanent magnets or electromagnets by considering different sizes of manipulation systems with varying field strengths and gradients achieved through the use of permanent magnets or electromagnets [62]. Besides, the manipulation of chiral skyrmions in Cu_2_OSeO_3_ was demonstrated under the influence of a magnetic field gradient, where the field gradient was used to control the motion of the skyrmions in a shift-register-like fashion, allowing for their use as information carriers in racetrack memory without the need for local electric currents. The skyrmions were found to rotate collectively in a radial field gradient, leading to a shell-like structure of discrete circular racetracks [63].

Another study proposed magnetic navigation system (MNS) for the wireless manipulation of micro-robots in human blood vessels consists of one conventional pair of Maxwell and Helmholtz coils and one newly developed pair of gradient and uniform saddle coils, which is theoretically developed using the Biot-Savart law and experimentally verified to be geometrically compact and magnetically efficient compared to the conventional MNS. The magnetic field gradient generated by the gradient and uniform saddle coils enables precise control of the micro-robots in the blood vessels [64]. Furthermore, the magnetization of the superparamagnetic nanoparticles, together with mutual interactions, led to the formation of aggregates in the direction of the homogeneous field, and the magnetic gradients introduced by the electromagnet directed the aggregates to the desired direction, while avoiding the formation of thrombosis, which is important for the thrombolysis with magnetic nanoparticles [65].

In short, the thrombolytic agents combined with MNPs could chemically and mechanically destroy the clot and reduce the hemorrhage risks and systemic toxicity, therefore achieving a minimally invasive way to treat thrombosis disease.

## III. APPLICATIONS OF MNPS IN THROMBOLYSIS

### A. IN VITRO THROMBOLYSIS IN A STATIC MAGNETIC FIELD

Many *in vitro* studies have been explored to increase the thrombolysis efficiency by manipulating thrombolytic drugs-loaded MNPs with a permanent magnet to reach the targeted thrombus location. Around 30 years ago, researchers found that by applying magnetic force, the urokinase-coupled magnetic modifier could be attracted to the fibrin gel and achieve local fibrinolysis [66]. Similar results were obtained in a later study with magnetic urokinase, which was more stable than the native enzyme in plasma and could be selectively transferred to fibrin clots by magnetic force [67].

Following this, different materials have been explored to increase the drug encapsulation rate of MNPs to lower the concentration of MNPs while maintaining a certain level of drug doses. MNPs can be coated with a biocompatible shell to prevent enzyme inactivation. For example, to deliver thrombolytic medicines to local thrombus, biodegradable magnetic microspheres containing tissue plasminogen activator (tPA) and magnetic iron oxide nanoparticles with polylactic acid-polyethylene glycol (PLA-PEG) copolymer coatings have been developed [68]. The advantage of this approach is that the magnetic iron oxide allows for the precise localization of the tPA to the thrombosis site, avoiding potential side effects associated with systemic administration of the drug. Moreover, the biodegradable microspheres containing tPA and magnetic iron oxide are superparamagnetic, meaning that they exhibit magnetic properties only in the presence of an external magnetic field, which is ideal for the targeted delivery of drugs. The high encapsulation and release of tPA from the microspheres in solution is also advantageous, as the tPA concentration (5.3–19.6 μg/mL) exceeds the theoretical thrombolysis concentration. To further improve the drug delivery efficiency, magnetic nanoparticles modified with carboxymethyl dextran (CMD) were designed to enhance the thrombolytic activity of tPA [69]. The study found that the optimum drug loading was achieved by contacting 0.25 mg rtPA with 5 mg CMD-MNP, where all rtPA is immobilized to the magnetic nanocarrier with full retention of its thrombolytic activity. In addition, different types of enzymes have been studied to demonstrate the potential of using magnetic nanoparticle conjugates for improved thrombolysis. For instance, a recent study aimed to immobilize two thrombolytic enzymes, nattokinase (NK) and lumbrukinase (LK), onto magnetic Fe_3_O_4_ nanoparticles for improved thrombolytic activity [70]. The results showed that the immobilized enzymes on the nanoparticles had much higher thrombolytic activity compared to the pure enzymes. The best thrombolytic activity was achieved with LK-nanoparticle conjugates (207.74%) compared to pure LK (106.57%), and NK-nanoparticle conjugates (91.89%) compared to pure NK (82.86%). Another interesting study confirmed that the tPA and streptokinase could also be conjugated with silica coated MNPs. The result demonstrated that magnetic targeting could achieve a 38 % reduction in blood clot lysis time than administering SiO_2_-MNPs without magnetic guidance, which revealed the potential for improved clinical thrombolytic therapy [71].

**FIGURE 5.**
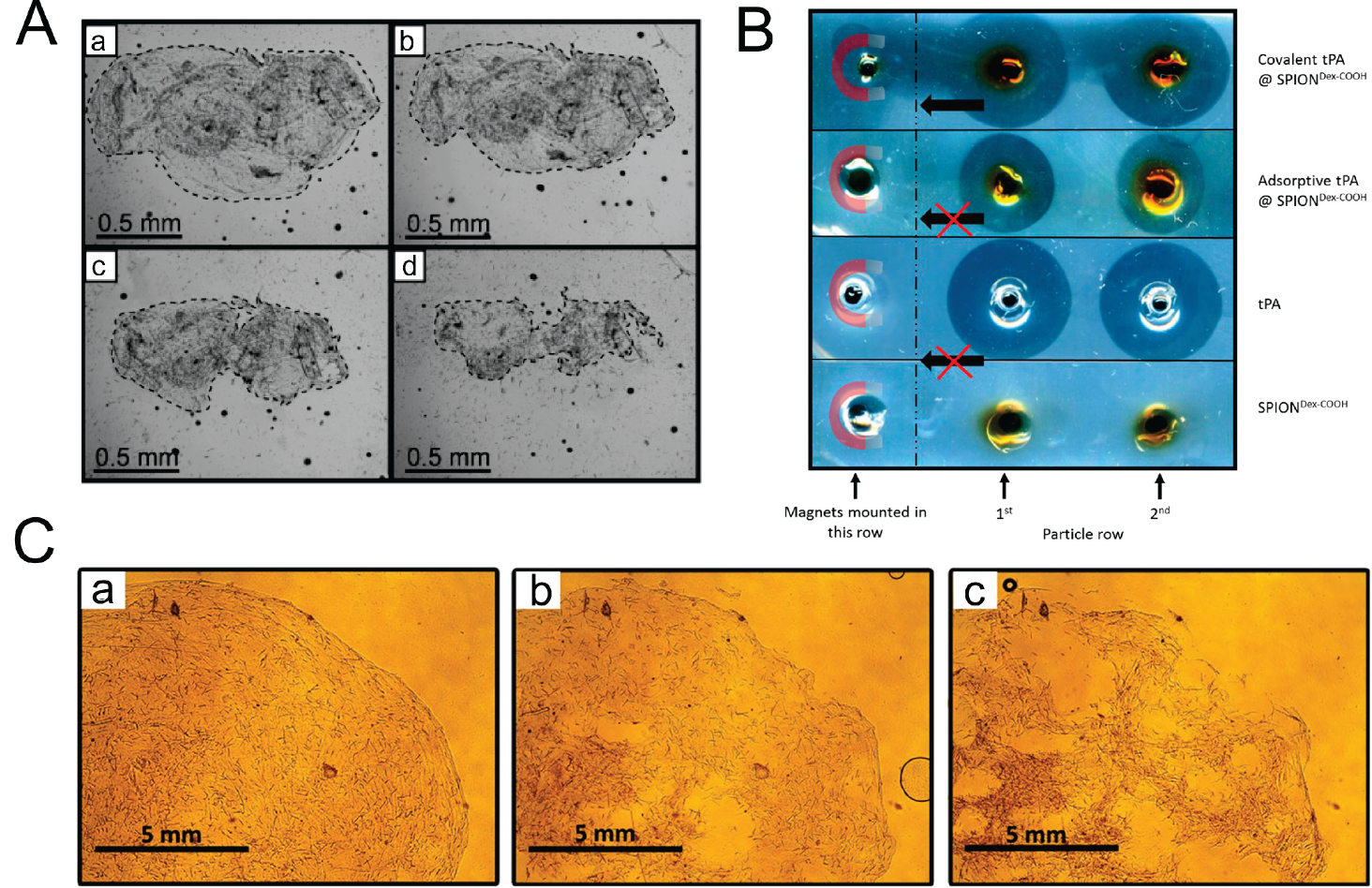
*In-vitro* thrombolysis with magnetic nanoparticles in a static magnetic field. (A) Targeted tPA@MNCs nanoparticles induce thrombolysis after treatments of 60, 245, and 620 minutes in a static magnetic field. The findings are as follows: (a) immediately after the tPA@MNCs are delivered to the thrombus site by the magnet, noticeable changes in the thrombus periphery are observed. This indicates that the tPA@MNCs have successfully reached the targeted area and are beginning to interact with the thrombus. (b) After 60 minutes, further changes in the thrombus are observed, suggesting that the tPA is being released from the nanocontainers and is initiating the process of clot breakdown. (c) At 245 minutes, additional progress in thrombolysis is seen, with the clot continuing to break down due to the ongoing activities of the released tPA. (d). Finally, after 620 minutes (approximately 10 hours) under static conditions, the thrombus is completely destroyed. Reproduced from [74], Copyright 2018, with the permission of IEEE. (B) The covalently bonded tPA on SPION^DexCOOH^ can dissolve agarose-fibrin matrices efficiently in the direction of the external magnetic field and the adsorptive method can only dissolve around the holes. Reproduced from [72], Copyright 2017, with the permission of IEEE. (C) Visualization of the plasma clot lysis process provided by magnetic thrombolytic composite using an optical microscope at 0 minutes, 45 minutes, and 90 minutes (a)–(c), X10 magnification. This fragmentation is characterized by an increase in the contact area between the surface of plasmin and the fibrin network, which leads to faster thrombolysis over time. Reproduced from [75], Copyright 2016, with the permission of IEEE.

Many recent studies have been focused on the drug-loaded magnetic iron oxide nanoparticles that can target blood clots by using a static magnetic field and induce local fibrinolysis. For example, as shown in Fig. 5(B), a magnetically guided drug delivery system was proposed for thrombolytic therapy using tPA-loaded dextran-coated MNPs, which presented no toxicity with primary human endothelial cells and exhibited high fibrinolytic activity in thrombus-mimicking fibrin-containing agarose gels [72]. However, coating approaches, on the other hand, may have issues with limited enzyme activity due to unintended release during drug delivery. Several creative solutions have been proposed to enclose enzymes in a nano or micro container to maintain their functionalities. For example, a recent study demonstrated that the streptokinase could be entrapped into a magnetite sol-gel matrix to create a magnetic targetable solid-state thrombolytic agent with non-releasing behavior for prolonged thrombolytic therapy [73]. The release of SK is controlled and delayed by the nature of its entrapment in the matrix, which prevents the enzyme from directly leaching into the surrounding medium. Instead, the nanoparticles act as a platform for SK activation and the formation of the plasmin-SK complex, which initiates clot dissolution. It is observed that when the mass fraction of entrapped streptokinase is below 10% wt., there is only a minor release of about 1% occurs, which confirms that the magnetite matrix is effective in controlling the release of the enzyme, contributing to the non-releasing behavior of the nanoparticles. The average time for lysis to start with free SK is 5 minutes, while for the SK@magnetite composites, it ranges from 30 minutes (12.5% concentration) to 420 minutes (5% concentration). This indicates that the controlled release of the enzyme results in a slower onset of action. The delay in release and the prolonged effect of the thrombolytic agent can be advantageous in clinical settings, as it allows for a more targeted and sustained thrombolytic therapy with reduced potential side effects. The magnetite matrix plays a crucial role in managing the release of SK and ensuring the effectiveness of these nanoparticles in clot dissolution. In the meantime, a different approach has been developed that shows promise for magnetically controlled thrombolysis, as shown in Fig. 5(A), where biocompatible tPA-loaded magnetite nanocontainers have been synthesized by indirect gelation of stable magnetite hydrosol at room temperature using the microemulsion method [74]. The figure shows a thrombolysis process using targeted tPA-loaded magnetite nanocontainers (tPA@MNCs) containing 10% tPA by weight. The process is monitored at various time intervals after the delivery of the containers to the thrombus site using a magnet. While the exact mechanism for releasing tPA from the nanocontainers under the influence of a magnetic field might not be clearly explored, it is possible that the magnetic field could either cause the nanocontainers to release tPA directly or trigger a change in the nanocontainer structure to allow for controlled tPA release. Besides, recent research has demonstrated the synthesis of thrombolytic magnetic composite material with non-releasing characteristic and extended activity, which results in faster thrombolysis over time [75]. As shown in Fig. 5(C), the process of clot lysis over time (0, 45, 90 minutes), with the clot gradually breaking down and becoming fragmented. This fragmentation is characterized by an increase in the contact area between the surface of plasmin and the fibrin network, which leads to faster thrombolysis over time. These results demonstrate the effectiveness of the magnetic thrombolytic composite in promoting the decomposition of blood clots and suggest that this technology may have potential applications in the treatment of thrombotic diseases. However, further research is needed to fully understand the safety and efficacy of this approach and to optimize the formulation of the magnetic thrombolytic composite for clinical use.

In addition, a pH-responsive coating, such as polyelectrolyte, can control drug release, leading to enhanced drug delivery efficiency. For example, the cationic polyelectrolyte grafted mesoporous magnetic silica composite particles with conjugated streptokinase was developed to enhance the thrombolytic activity for targeted thrombolysis treatment [76]. The composite particles display a pH-dependent phase transition near 5.8. In the presence of 11.2 kU/mg conjugated streptokinase (SK), the Fe_3_O_4_/SiO_2_/P(iBMA-APTMACl) composite particles exhibit a significant thrombolytic activity of 56% under magnetic targeting. A brief hemolysis study suggests that the composite particles are hemocompatible up to 0.18 mg/mL. Besides, the higher inhibition percentage of protein denaturation indicates the anti-inflammatory property of the bare composite particles. The prepared composite particles show potential for application in targeted thrombolysis, drug/gene carrier systems, anti-inflammatory agents, and hyperthermia treatment. Furthermore, a recent study demonstrated that urokinase-conjugated magnetic polyelectrolyte-based composites with dual anticoagulant and thrombolytic properties could significantly increase the clot lysis rate [77]. The study showed the development of four magnetic thrombolytic systems with urokinase-type plasminogen activator (uPA) conjugated to a magnetite core via different polyelectrolyte molecules, showing varying degrees of thrombolytic and anticoagulant activities, with the heparin-based system demonstrating the highest thrombolytic activity and moderate anticoagulant activity. Carboxymethyl chitosan (CMC) and sodium alginate (SA) are two typical ingredients used to modify MNPs using the co-precipitation approach to optimize the sustained drug release effects. For instance, nattokinase-conjugated magnetite nanoparticles have been prepared with the adjunct of CMC and SA for improved thrombolytic efficacy with prolonged drug release and magnetic targeting [78]. This study demonstrated the development of NK-conjugated magnetite nanoparticles for accurate delivery of NK to the thrombus site, which showed sustained release thrombolysis potential, magnetic targeting capability, and storage stability, indicating potential for improved thrombolytic efficacy. The use of targeted drug delivery systems like NK-MNPs has the potential to enhance the therapeutic effectiveness of thrombolytic agents and reduce side effects by delivering the drug specifically to the site of thrombus. Further studies are needed to evaluate the *in vivo* efficacy and safety of NK-MNPs for clinical translation. Also, a novel application of capturing and dissolving clots using streptokinase-loaded ferrite zinc MNPs synthesized via the co-precipitation method was demonstrated in a flow model [79]. The study evaluated the efficiency of nano-magnetic capturing and dissolving of clots (NCDC) by exposing different levels of process parameters, including magnetic field strength and fluid flow rate, to different sizes of blood clots in an *in vitro* flow model. The results showed that increasing the parameters to their maximum values could immobilize 100% of the clots and dissolve around 61.4% of the clots. The efficiency of NCDC is directly proportional to the magnetic field strength. However, it is important to note that the study was conducted *in vitro*, and further research is needed to evaluate the effectiveness and safety of NCDC *in vivo*.

In summary, as shown in Table 1, many *in vitro* studies demonstrated that MNPs combined with various thrombolytic agents could enhance the thrombolysis rate under a static magnetic field while maintaining a low dose of drugs (a smaller amount of the thrombolytic drug is used compared to the standard or typical dose used for thrombolysis), which helps treat many thrombosis diseases such as strokes and deep vein thrombosis, and pulmonary embolism. The standard dose of thrombolytic drugs varies depending on the specific drug being used and the condition being treated. For example, the standard dose of alteplase (also known as tissue plasminogen activator or tPA) for the treatment of acute ischemic stroke is 0.9 mg per kilogram of body weight, up to a maximum dose of 90 mg, administered intravenously over the course of one hour [80]. The standard dose of Tenecteplase (also a thrombolytic drug) for the treatment of myocardial infarction is 30–50 mg administered intravenously over 5–10 seconds [81].

### B. IN-VIVO THROMBOLYSIS IN A STATIC MAGNETIC FIELD

The feasibility of thrombolysis with MNPs was also demonstrated in various *in-vivo* studies. Since the 1980s, researchers have demonstrated that MNPs with immobilized streptokinase could be injected intravenously into canine arteries and induce complete thrombus degradation and regular blood flow restoration under external samarium-cobalt magnet guidance [82]. However, the small sample size (n = 2) could potentially lead to bias and limits the ability to draw definitive conclusions of this study. Additionally, the study lacks information on potential side effects or toxicity of the magnetic carrier system and further studies would be needed to address these concerns. Various *in-vivo* studies have been explored in a rat model following this insight. For example, magnetic drug targeting in a rat embolic model has been evaluated using MNPs for effective retention against hemodynamic dragging force in the iliac artery [83]. The study involves injecting a whole blood clot *in vitro* from the right iliac artery and lodging it in the left iliac artery of anesthetized rats. Subsequently, recombinant tissue plasminogen activator (rt-PA) is administered intra-arterially to reverse the iliac flow within 15 minutes. The placement of a NdFeB magnet above the left iliac artery causes magnetic nanoparticle retention against hemodynamic dragging force, both in the presence and absence of the clot. The use of magnetic nanoparticles for the targeted delivery of thrombolytic drugs is a promising approach to reducing the side effects of conventional thrombolytic drug administration. However, there are still many challenges to be overcome before this approach can be effectively used in clinical settings. These include optimizing the magnetic targeting system and addressing potential toxicity and immunogenicity concerns associated with the use of magnetic nanoparticles. Nonetheless, this study represents an important step toward the development of safer and more effective thrombolytic drug delivery systems. Besides, immobilization of tPA to MNP by covalent bonding may improve the concentration and activity of tPA near the clot in the presence of blood flow. For instance, polyacrylic acid-coated magnetite with tPA shows effective target thrombolysis under magnetic guidance [84]. The enzyme activities of bound rtPA were found to be 87% and 86% of free rtPA, as measured by chromogenic substrate assay and 125I-fibrinolysis assay. The study used a rat embolic model to evaluate the thrombolytic activity of PAA-MNP-rtPA with rtPA equivalent to 0.2 mg/kg. Under magnetic guidance with the magnet moving back and forth along the iliac artery, intra-arterial administration of PAA-MNP-rtPA restored the iliac blood flow within 75 minutes to 82% of that before the clot lodging. This approach may achieve reproducible and effective target thrombolysis with less than 20% of a regular dose of rtPA. The use of magnetic nanoparticles and targeted drug delivery could potentially reduce the risk of hemorrhagic side effects associated with conventional thrombolytic drug administration. However, the amount of tPA loaded per MNP may be limited, necessitating a greater dose to attain therapeutic concentration at the clot site of interest. Further research is needed to evaluate the safety and efficacy of this approach in larger animal models and clinical trials.

Therefore, various coating materials, such as dextran, poly[aniline-co-N-(1-one-butyric acid) aniline] (SPAnH), chitosan, and silica have been used in the development of biocompatible MNPs with a greater capacity for drug loading. For example, the improved thrombolysis efficiency of urokinase-conjugated MNPs with dextran coating was demonstrated in a rat arteriovenous shunt thrombosis model under magnetic guidance [85]. The study found that the magnetic field generated by two AlNiCo permanent magnets around the site of the thrombus enhanced the thrombolytic efficacy of the conjugate by 5-fold over urokinase with no reduction in plasma fibrinogen and little prolonged bleeding time. The targeting efficiency could be instantly improved by increasing the strength of the magnetic field or by increasing the magnetization values of MNPs carriers. This study provides compelling evidence for the potential of magnetically targeted thrombolysis to be a safe and effective treatment option for thrombosis. The use of MNPs to deliver thrombolytics to specific sites could minimize the risk of severe bleeding associated with traditional thrombolytic therapies. It is also important to consider the optimal magnetic field strength and MNPs carrier properties to ensure maximal thrombolytic efficacy while avoiding adverse effects. In addition, the low-toxicity magnetic nanocarriers with immobilized tPA loaded in the SPAnH shells showed the successful restoration of blood flow within 15–25 minutes of therapy in a rat embolism model [86]. *In vitro* thrombolysis testing with a tubing system demonstrated that magnet-guided MNC-rtPA showed significantly improved thrombolysis compared with free rtPA and reduced the clot lysis time from 39.2 ± 3.2 minutes to 10.8 ± 4.2 minutes. The development of low-toxicity magnetic nanocarriers for targeted thrombolysis is a promising approach to reducing the risk of hemorrhagic side effects associated with traditional thrombolytic therapies. In another study, chitosan coated MNPs were covalently linked to tPA for targeted thrombolysis in a rat embolic model. The optimum drug loading was reached when 0.5 mg tPA was conjugated with 5 mg chitosan-MNP, where 95% of the drug was attached to the carrier with full retention of its thrombolytic activity. Under magnetic guidance, chitosan-MNP-tPA reduced the blood clot lysis time by 58% compared with runs without magnetic targeting or by 53% compared with free tPA using the same drug dosage. Effective thrombolysis in response to chitosan-MNP-tPA under magnetic guidance was also demonstrated in a rat embolic model where one-fifth of the dose of tPA exerted similar thrombolytic efficacy of the drug [87]. The significant reduction in blood clot lysis time under magnetic guidance compared with runs without magnetic targeting or with free tPA using the same drug dosage further supports the potential of this approach. In a further study, silica-coated tPA-conjugated MNPs with increased drug loading capability were prepared and demonstrated effective thrombolysis under magnetic control in an *ex-vivo* thrombolysis model [88]. When 0.5 mg/mL tPA is conjugated with 5 mg SiO_2_-MNP, the optimal drug loading is achieved, with 94% of tPA attached to the carrier, 86% of amidolytic activity retained, and full fibrinolytic activity retained. The excellent biocompatibility and enhanced storage and operational stability of tPA after conjugation with SiO_2_-MNP further support the potential of this approach. The effective thrombolysis under magnetic guidance in an *ex vivo* model provides evidence for the feasibility of this approach.

**FIGURE 6.**
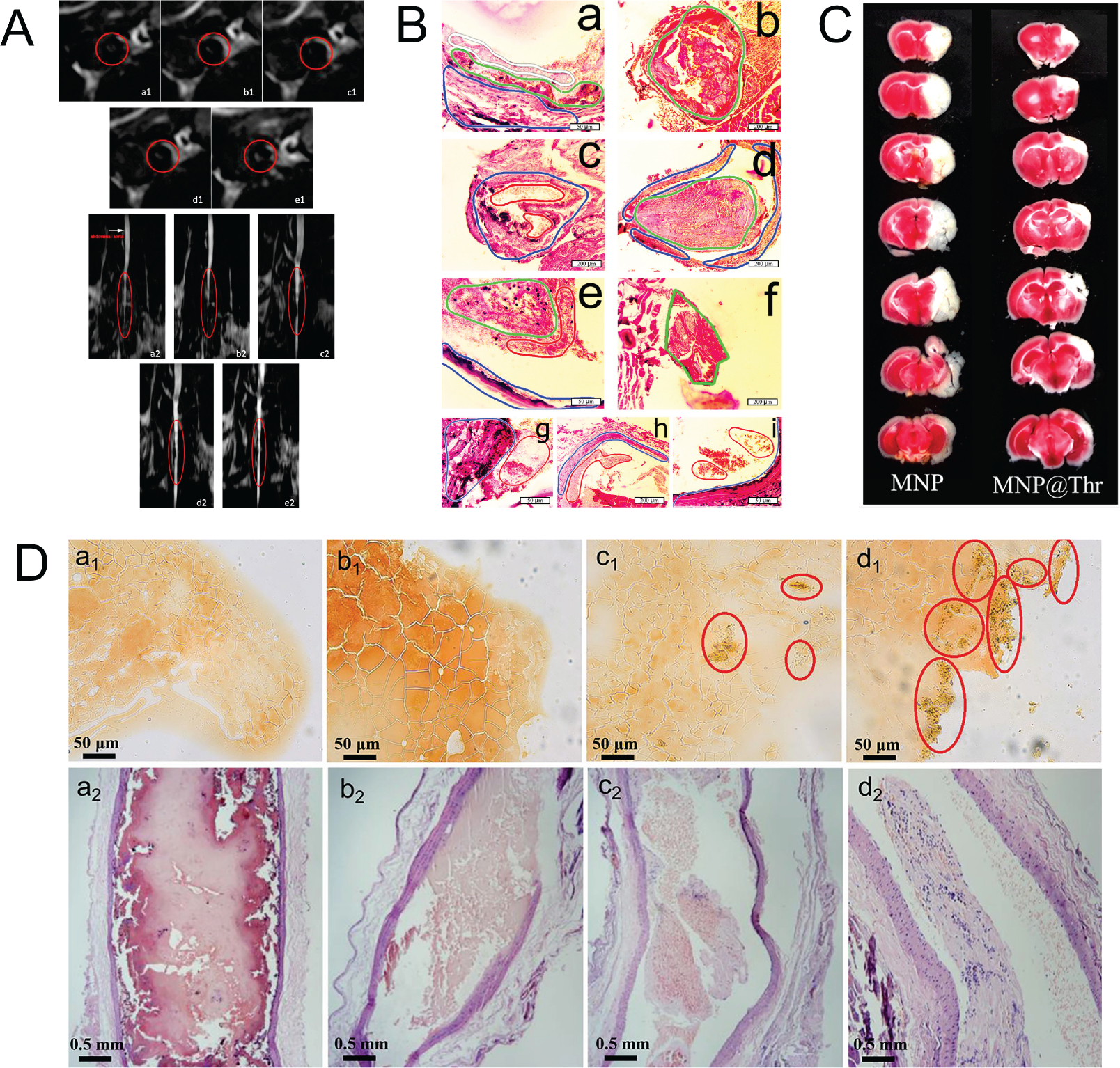
*In-vivo* thrombolysis with magnetic nanoparticles in a static magnetic field. (A) The study used T2-weighted and Vs3DI imaging techniques to monitor the in vivo thrombolysis after abdominal aorta injuries using MR. The treatment area (a1), (a2) was imaged before and after treatment with Fe3O4−PLGA-rtPA/CS-cRGD nanoparticles at 10 (b1), (b2), 20 (c1), (c2), 40 (d1), (d2), and 60 (e1), (e2) min. The T2-weighted images showed a decrease in the T2 signal at the mural thrombus and a widening of the hypointensity zone (red circles) at 10 and 20 min after injection. The Vs3DI images showed the formation of a thrombus in the rat abdominal aorta, and partial reappearance of the vessel lumen signal (red circles) was detected at 40 and 60 min after treatment with the nanoparticles. Reproduced from [90], Copyright 2014, with the permission of IEEE. (B) The results of histological examination of animals injected with saline, uPA, or MNPs@uPA. In the rat carotid artery, the saline group showed a white parietal clot containing multiple aggregates of adhered erythrocytes, dense fibrin, platelets, and leukocytes. In contrast, only single adherent erythrocytes near the inner wall of the vessel were observed in animals injected with MNPs@uPA, while multiple large erythrocyte aggregates completely covered the vessel wall and occupied >50% of the lumen in animals injected with uPA. Similar findings were observed in the rabbit femoral artery. (a)–(f) Sections of the rat carotid artery and (g)–(i) rabbit femoral artery 24 h post clot formation. Colors: blue, vessel walls; red, erythrocyte aggregates; green, red clot; white, white clot. Reproduced from [92], Copyright 2018, with the permission of IEEE. (C) The impact of thrombolytic drug on the stroke model induced by MNP@Thrombin was investigated. The changes in the infarction size with MNP or MNP@Thrombin after thrombolytic treatment were observed through TTC staining images. TTC staining, which can identify neuronal degradation, was employed to detect any pathological alterations after stroke. The use of U.K. led to a decrease in stroke lesions’ sizes and reduced neuronal degradation. These results were in line with the clinical scenario. Reproduced from [99], Copyright 2021, with the permission of IEEE. (D) The study aimed to investigate the dual targeting of Fe_3_O_4_-(4-PLA(G_3_)_4_)-RGD/nattokinase nanoparticles in male rats using RGD and magnetic targeting. After inducing thrombi formation, the rats were administered with 500 *μ*L of PBS or PBS with either nattokinase or Fe_3_O_4_-(4-PLA(G_3_)_4_)-RGD/nattokinase through the tail vein. Frozen sections of the thrombi were observed and labeled as 1, with the Fe_3_O_4_-(4-PLA(G_3_)_4_)-RGD/nattokinase nanoparticles marked in a red circle. Hematoxylin and eosin staining of the experimental vessels was also performed and labeled as 2, after intravenous injection of PBS, PBS with nattokinase, PBS with Fe_3_O_4_-(4-PLA(G_3_)_4_)-RGD/nattokinase, and PBS with Fe_3_O_4_-(4-PLA(G_3_)_4_)-RGD/nattokinase under an external magnet. Reproduced from [95], Copyright 2020, with the permission of IEEE.

Moreover, numerous methods, such as micro-computed tomography (micro-CT), MRI scan, blood pressure and flow monitoring, fluorescence imaging, and infrared thermal imaging, have been utilized to assess the efficacy of thrombolysis in animal tests. For example, a drug retention assessment study based on the micro-CT image of rat arteries showed that intravenous administration of tPA-loaded MNPs could maintain high concentrations at local thrombus to achieve effective thrombolysis [89]. Effective thrombolysis is demonstrated in an *ex vivo* model with magnetic targeting, showing 34% and 40% reductions in blood clot lysis time compared to runs without magnetic targeting and with free tPA, respectively. Microcomputed tomography analysis confirms enhanced penetration of SiO_2_-MNP-tPA into blood clots under magnetic guidance. In addition, as shown in Fig. 6(A), the MRI scan showed that tPA-conjugated MNPs could accumulate on the clot surface and significantly improved the thrombolysis efficiency compared to the tPA alone case [90]. *In vitro* and *in vivo* experiments showed that the Fe_3_O_4_-PLGA-rtPA/CS-cRGD nanoparticles had a significant effect on thrombolysis compared to other nanoparticles and free rtPA solution. The study highlights the potential of these nanoparticles as a dual-function tool in the early detection of thrombi and in the dynamic monitoring of thrombolytic efficiency using MRI at the molecular level. In another investigation, using magnetic guidance, blood pressure and flow were measured before and after the delivery of tPA and streptokinase conjugated MNPs in a rat embolism model. Effective thrombolysis with magnet-guided SiO_2_-CuNPs-tPA-SK was demonstrated in a rat embolism model, with 18.6% of the regular t-PA dose and 15.78% of SK dose restored and 15–25 min reductions in blood clot lysis time compared to runs with free t-PA and without magnet guidance using the same drug dosage [91]. However, further studies are needed to assess the safety and efficacy of this method, including long-term toxicity studies and clinical trials. Additionally, the study did not fully address the comparison between CuNPs and MNPs for thrombolysis, leaving room for future research to determine the best carrier type for targeted drug delivery in thrombotic disease. Besides, as shown in Fig. 6(B), a potential treatment of blood clotting was evaluated by fluorescence imaging in rat carotid artery and rabbit femoral artery with novel magnetic nanocomposite prepared by heparin-mediated cross-linking of urokinase with magnetite nanoparticles [92]. The study also showed that magnetic control of MNPs@uPA positioning increased its thrombolytic efficacy by at least 30%. Importantly, the composition was found to be biocompatible, non-toxic, and safe in rats and rabbits, even at concentrations that exceeded the therapeutic dose by 3 orders of magnitude. However, the study was limited to *in vitro* and animal experiments and further studies are required to assess the safety and efficacy of the composition in humans. In a recent study, infrared thermal imaging was used to evaluate the thrombolysis treatment by intravenous administration of tPA-loaded thermosensitive magneto-liposome (TML@rtPA) in a rat embolic model under magnetic steering and focal hyperthermia dual-controlled therapy [93]. The release of rtPA equivalent to 20% regular dose from TML@rtPA administered intra-arterial vs. intravenous (i.v.) significantly restored iliac blood flow 15 vs. 55 min after clot lodging, respectively. The study presents promising results, indicating that TML@rtPA can achieve target thrombolysis and restore blood flow more quickly than traditional i.v. administration. However, it is important to note that this was a preclinical study conducted on rats, and further research is necessary to determine the safety and efficacy of this approach in human subjects. Additionally, the study only tested a single focal spot on the iliac artery, and it is unclear how this approach would perform in more complex or widespread thrombotic events.

In addition to the above research, many studies have been focused on improving drug stability and the MNPs targeting efficiency using a dual targeting strategy for the animal test. For example, using dual-targeted therapy, thrombus-targeting nanocomposites loaded with nattokinase demonstrated significant thrombolysis ability [94]. The *in vivo* thrombolytic effect was observed to be the best for M-MSNs-G_3_-RGD/NK under the local application of a magnet, which dissolved the thrombus into small pieces. Further studies are required to confirm the efficacy and safety of M-MSNs-G_3_-RGD/NK in larger animal models. Additionally, the study does not provide information on the long-term stability and degradation of the nanoparticles, which is an important consideration for clinical translation. Comparable results were obtained in a later study with nattokinase-conjugated MNPs and RGD-modified dendrimers for dual-targeting thrombolysis treatment, as shown in Fig. 6(D) [95]. The results of *in vitro* and *in vivo* experiments showed that Fe_3_O_4_-(4-PLA(G_3_)_4_)-RGD/nattokinase provided a higher blood clot dissolution rate compared to free nattokinase, and most of the thrombi were dissolved under an external magnetic field. Additionally, the nanoparticles were found in vascular endothelial cells, demonstrating the RGD and magnetic dual targeting capacity of Fe_3_O_4_-(4-PLA(G_3_)_4_)-RGD/nattokinase. One potential limitation of this study is that the long-term toxicity was not evaluated, so the safety of long-term use remains unknown. Peptide and tPA-conjugated poly (lactic-co-glycolic acid) (PLGA) MNPs have also been prepared for dual-targeted thrombolysis in a rat embolic model [96]. The strategy involves co-immobilizing rtPA and fibrin-avid peptide to magnetic nanoparticles (PMNP) and preparing peptide/rtPA conjugated PMNPs (pPMNP-rtPA) using surface modification with avidin and biotin-PEG-rtPA (or biotin-PEG-peptide). The pPMNP-rtPA could reduce the clot lysis time for reperfusion by 40% when compared to free rtPA at the same drug dosage in the pressure-dependent clot lysis model in a flow system. In the rat embolic model, pPMNPrtPA was used at 20% of free rtPA dosage to restore the iliac blood flow in vascular thrombus. However, some limitations should be noted such as the stability and long-term toxicity of pPMNP-rtPA should be carefully evaluated and the fabrication process of pPMNP-rtPA is complex and involves multiple steps, which may affect the reproducibility and scalability of the nanoparticles.

In contrast to the thrombolytic drugs mentioned above, another work demonstrated the feasibility of heparin targeting delivery with polyethyleneimine (PEI) modified magnetic black phosphorus nanosheets for increased heparin loading capacity and prolonged thromboprophylaxis treatment [97]. The platform was found to have high heparin loading capacity of approximately 450%, accurate magnetic enrichment capacity, and good biocompatibility both *in vitro* and *in vivo*. The use of NIR laser irradiation further enhanced the photothermal thrombolysis effect. However, the study lacks a direct comparison with existing thromboprophylaxis drugs and delivery systems, making it difficult to assess the potential impact and advantages of this platform in the field. Moreover, the effect of PEGylation has been investigated to improve the half-life and stability of dextran-coated MNPs under magnetic capture, which may enhance the therapeutic efficacy [98]. The study showed that magnet-induced retention of 250 nm MNPs was associated with a variable reduction in RBC flow, suggesting a dynamic coupling of hemodynamic and magnetic forces. PEGylation of the MNPs resulted in faster restoration of flow after magnet removal, possibly due to a reduced interaction with vascular endothelium. However, further studies are needed to determine the potential impact of PEGylation on the overall pharmacokinetics and therapeutic efficacy of the nanoparticles. Besides, as shown in Fig. 6(C), the thrombin cross-linked MNPs were prepared to help thrombus formation in the rat carotid artery and provide an easy and minimally invasive way for the new stroke model [99]. The method involves positioning a magnet in the common carotid artery and injecting the MNP@Thrombin from the tail vein. The MNP@Thrombin then accumulates in the carotid artery and induces thrombus formation, which subsequently blocks the cerebral artery. The study showed that the model responds well to thrombolytic drugs and can be used for pharmacological and rehabilitative research. However, further studies are needed to assess the reproducibility and reliability of this model and may not be directly transferable to humans.

Meanwhile, the feasibility of magnetic drug targeting was also demonstrated in some large animal models. For example, the magnetic tPA carriers could be injected intravenously and trigger the release of thrombolytic drugs using an external magnetic field at the clot site in large arteries of primate models (monkeys) [100]. The results showed intra-arterial carrier trapping selectively at the arterial region exposed to an external magnetic field. The study provides a theoretical framework and preliminary experimental data on the feasibility of magnetic trapping of blood-borne magnetic nano- and microcarriers in human large vessels, especially arteries. However, the study lacks detailed information on the preparation and characterization of t-PA-loaded magnetic nano- and microcarriers and the design of magnetic guidance systems. More detailed studies are required to establish the safety, efficacy, and translational potential of this approach before it can be translated into clinical practice. In another study, the treatment of in-stent thrombolysis with tPA-conjugated MNPs has been demonstrated in coronary arteries with a pig model [101]. *In vivo* biocompatibility tests in pigs showed no short-term adverse effects of intravascular injection of the nanoparticles in a stented brachial artery. Another pig experiment showed successful implant-assisted magnetic drug targeting through the action of the thrombolytic drug immobilized to the nanoparticles. The results suggest that a substantially lower dose of tPA may be used for the lysis of a thrombus when the drug is immobilized to magnetic nanoparticles and targeted to the thrombus by implant-assisted magnetic targeting. However, the study was limited by its small sample size, and more animal studies are needed to verify a statistically significant effect.

Table 2 summarizes studies where the static magnetic field was applied to retain and manipulate MNPs in animal vessels. The thrombolytic drug-loaded MNPs under magnetic guidance may enhance the thrombolysis efficacy with low-dosage drugs and improve clinical outcomes.

However, concerns about the biosafety and biometabolism of MNPs have also been raised, particularly in the context of *in vivo* applications [102]. Ferrite nanoparticles, such as iron oxide nanoparticles, are among the most widely used MNPs for *in vivo* applications, including thrombolysis [85], [103]. These nanoparticles have been shown to exhibit low toxicity and high biocompatibility, and they can be metabolized and excreted through the liver and spleen [104], [105]. The surface of ferrite nanoparticles can also be modified with various coatings or functional groups to enhance their biocompatibility and reduce their toxicity [106], [107], [108]. For example, the use of polyethylene glycol (PEG) coatings can improve the stability and biocompatibility of iron oxide nanoparticles [98], [101], [108], [109]. Other types of MNPs, such as metallic nanoparticles and metallic nanoparticles with a shell, may have different biocompatibility and biometabolism profiles compared to ferrite nanoparticles. For example, some metallic nanoparticles may exhibit cytotoxicity or oxidative stress, which can be attributed to their high reactivity and potential for generating reactive oxygen species [110]. The surface coatings of metallic nanoparticles can also affect their biocompatibility and biometabolism, with some coatings such as cationic surfactants showing potential toxicity [111]. Overall, the biosafety and biometabolism of MNPs *in vivo* depend on various factors, including their composition, size, surface coating, and dose [112]. The use of biocompatible and biodegradable MNPs with minimal toxicity and efficient biometabolism is critical for the success and safety of *in vivo* thrombolysis and other biomedical applications. Further research is needed to optimize the design and synthesis of MNPs for improved biocompatibility and biometabolism, as well as to assess their long-term safety and potential for clinical translation.

### C. IN VITRO THROMBOLYSIS IN A DYNAMIC MAGNETIC FIELD

In addition to the static magnetic field guided magnetic nanoparticle thrombolysis, many *in vitro* studies have been focused on the improvement of thrombolysis efficacy by applying dynamic magnetic field such as a rotating permanent magnet to generate a rotating magnetic field (RMF) or applying alternating current into electromagnetic coils to generate an alternating magnetic field (AMF). For instance, the rotational flows developed by translational and rotational motions of MNPs under oscillating magnetic fields are demonstrated to remove the clot in blocked microchannels effectively [113]. The rotation-induced fluid flow ablated the occlusion and created a narrow pathway in the central area of the thrombus. The non-contact removal of the central thrombus area was completed in less than 30 seconds using a gradient of 2.54 × 10^6^ A/m^2^ and oscillating field strength of 624 A/m. The study presents an interesting approach for micro-surgery, but the results are limited to a microchannel model. Further studies are necessary to evaluate the efficacy of the method in more complex systems and *in vivo*.

**FIGURE 7.**
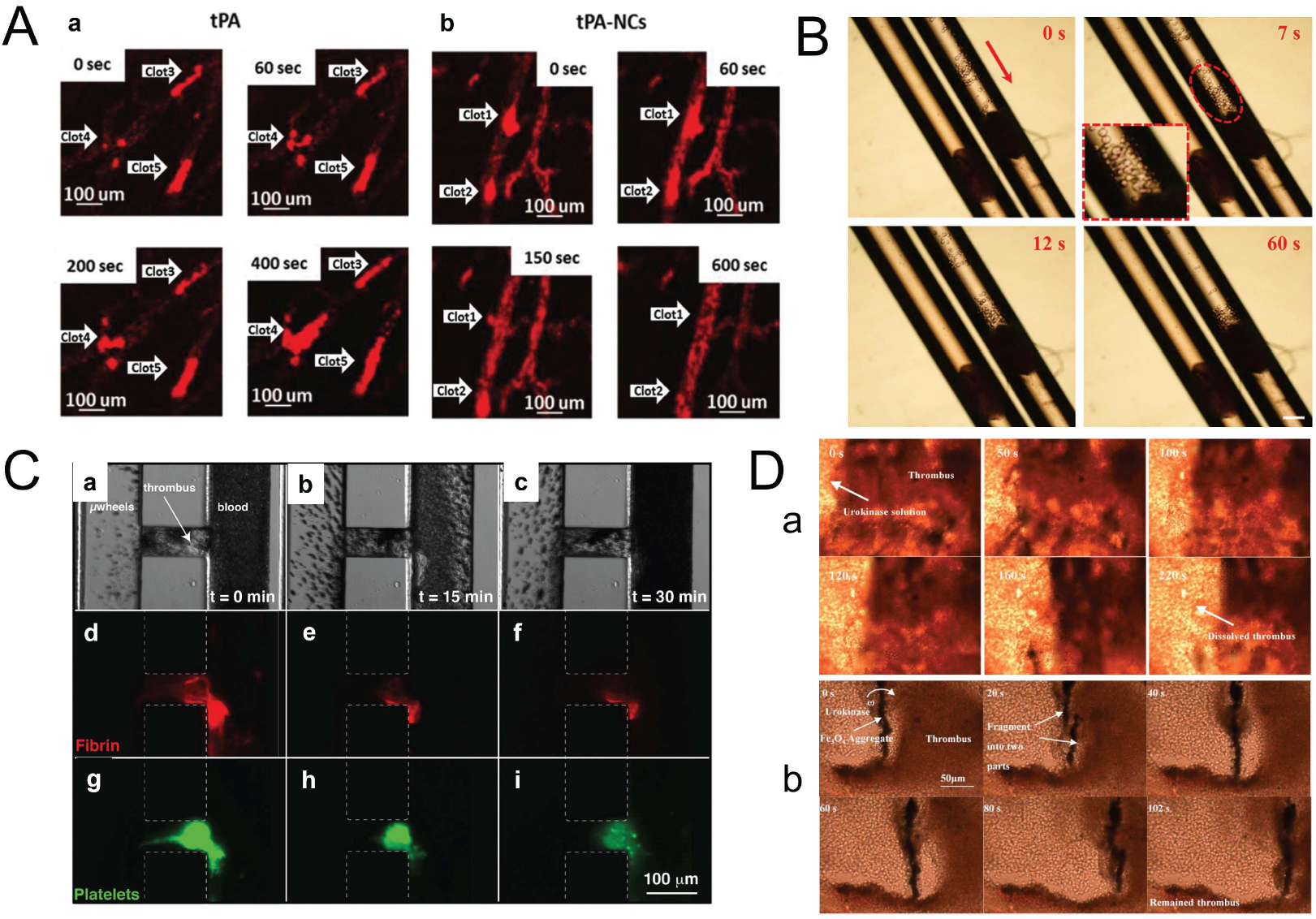
*In-vitro* thrombolysis with magnetic nanoparticles in a dynamic magnetic field. (A) During treatments with (a) free tPA and (b) tPA-NCs, representative intravital microscopy pictures of the mesentery vasculature were acquired at various times. Reproduced from [114], Copyright 2015, with the permission of IEEE. (B) BMMs provided a platform for microvascular thrombolysis that was extremely minimally invasive. BMM swarm is propelled toward a blood clot in an artificial glass vein. The tPA-loaded BMMs were activated to collectively target and concentrate at the site of the blood clot in artificial vasculature using a rotating magnetic field. Scale bar: 300 μm. Reproduced from [116], Copyright 2020, with the permission of IEEE. (C) A platelet-rich thrombus was treated in a microfluidic model of hemostasis by tPA-wheels. After the horizontal channel coated with collagen-mimetic peptides and tissue factor is occluded by the thrombus, μwheels are introduced from the left vertical channel while blood is present in the right vertical channel. a, b, and c show brightfield images of μwheels accumulating at and penetrating into the thrombus. d to f shows epifluorescence of fibrin(ogen), while g to i shows epifluorescence of platelets. Reproduced from [118], Copyright 2017, with the permission of IEEE. (D) (a) These image sequences demonstrate a thrombus removal process that was solely mediated by urokinase. The moving boundary of the thrombus indicates that the thrombus was gradually eliminated by the diffusion of the injected urokinase. The average speed of the boundary movement was approximately 20 μm/min. (b) The image sequences depict the process of thrombus removal using RMF-guided Fe_3_O_4_ NPs. In the experiment, urokinase at a concentration of 50 μg/mL and NPs at a concentration of 10 mg/mL were used, and the interval between images was 102 s. At the start of the experiment (0 s), the NPs were seen to cluster into a microrod with a length of about 150 μm under the influence of the static magnetic field. The microrod then began to rotate with an angular velocity of ω, generated by the RMF. This rotation produced a vortex, which enhanced the diffusion of urokinase to the surface of the thrombus and accelerated its dissolution. Although the cohesive force of the aggregate was not strong enough, causing it to fragment into two parts at times (as shown in the images at 20 s and 80 s), the phenomenon of enhanced diffusion of urokinase continued, and the 2 mg thrombus was ablated in about 102 s. The thrombolysis speed was approximately 36 μm/min, which is 1.8 times faster than using the same dose of pure urokinase alone. Reproduced from [55], Copyright 2018, with the permission of IEEE.

Many studies used thrombolytic drugs such as tPA and urokinase to increase the lysis rate in a dynamic magnetic field by combining different kinds of magnetic particles such as nanocubes, nanorods, liposomes, and micro-wheels. The dynamic magnetic field can induce temperature change which helps to control the drug release. For example, as shown in Fig. 7(A), the tPA-conjugated iron oxide nanocubes (tPA-NCs) were activated under alternating magnetic fields for enhanced thrombolysis by direct tPA contact with clot fibrin and localized hyperthermia effect [114]. The proposed nanoconstructs demonstrate a 100-fold increase in dissolution rate compared to free tPA *in vitro*, and a 10-fold enhancement when exposed to AMF. Intravital microscopy experiments demonstrate blood vessel reperfusion within a few minutes post tail vein injection of tPA-NCs. Moreover, the tPA-encapsulated thermosensitive magnetic liposomes were prepared for magnetically targeted delivery to the clot site and temperature-sensitive controlled release of the tPA drug under an alternating magnetic field [115]. The *in vitro* thrombolysis experiments confirmed the temperature-sensitive release of rtPA from TML, with a higher temperature resulting in a higher drug release rate. The thrombolytic activity of TML-rtPA at 43 °C was higher than at 37 °C, indicating that temperature-sensitive drug release at the thrombus site could be a feasible strategy for controlled release of rtPA in an alternating magnetic field *in vivo*. In another work, as shown in Fig. 7(B), bioinspired soft microrobots with precise magnetic control under a rotating magnetic field were developed for ultra-minimal invasive thrombolysis treatment with tPA [116]. The study developed a biomimetic magnetic microrobot (BMM) inspired by magnetotactic bacteria (MTB) for targeted thrombolysis. The BMM achieved a maximum speed of 161.7 μm/s and accurate positioning control under a rotating magnetic field with less than 4% deviation. The BMMs demonstrated frequency-dependent synchronization under 8 Hz and asynchronization at higher frequencies due to the increased drag torque. The BMM design inspired by MTB is unique and provides advantages in locomotion capacity and magnetic sensitivity over other magnetic microrobots. However, the study is limited to *in vitro* experiments, and further research is needed to evaluate the BMMs *in vivo*. Recently, MNPs with different morphology and dimensionality, such as chain-like 1D and sea urchins-like 3D structures, were demonstrated to be most effective for thrombolysis treatment of 3 days old clots [117]. The primary mechanism underlying the enhancement of enzymatic activity lies in the mechanical disturbance of fibrin mesh and intensified mass transfer in clot proximity. Both of these processes are closely related to the frequency of the external rotating magnetic field.

Additionally, the motion control of MNPs under dynamic magnetic may aid in improving the drug release and diffusion to the clot location. For example, as shown in Fig. 7(C), the magnetic colloidal micro-wheels were designed with the ability to assemble, rotate, translate and disassemble under magnetic fields excitation for the delivery of high local concentrations of tPA to induce thrombolysis treatment [118]. The fibrinolysis rate of plasma-derived fibrin gels by tPA-microwheels is fivefold faster than with 1 μg/mL tPA, and the microwheels can penetrate through 100 μm sized platelet-rich thrombi in about 5 min. The unique combination of surface and bulk dissolution mechanisms with mechanical action could provide a faster targeted fibrinolysis strategy than diffusion-limited approaches. However, the feasibility of using colloidal microwheels for relieving thrombosis in small vessels needs to be validated in future studies. Furthermore, the tPA-conjugated iron oxide nanorods have been developed to increase the release of tPA under external rotating magnetic fields to improve thrombolytic efficiency [119]. The nanorods were found to release tPA within ~30 minutes and could be controlled using an external rotating magnetic field, which enhanced the thrombolytic efficiency. *In vitro* thrombolysis assays showed that the nanorods achieved a lysis efficiency of 70%, significantly higher than the 30% achieved by tPA solution. However, the study has some limitations, such as the lack of *in vivo* testing and the potential issue of nanorod aggregation, which may block capillaries. Besides, magnetic nanoparticle movement and thrombolysis efficiency were remotely evaluated through biomimetic vascular channels, and the results showed enhanced tPA delivery and fibrinolysis activity in 85% of dynamic MNP + tPA experiments in rotating magnetic fields [120]. While this research presents an interesting and potentially useful approach to drug delivery, the study is limited to an *in vitro* model and further studies are needed to determine the safety and efficacy of MNPs in animal and human models. Additionally, the study does not address the potential risks and side effects associated with the use of MNPs.

Furthermore, several recent experiments with a dynamic magnetic field have also used urokinase instead of tPA. For example, the urokinase-coated MNPs were utilized to induce the micro-ablation of blood clots under an oscillating magnetic field in a microchannel [121]. The results showed that the use of urokinase-coated Fe_3_O_4_ nanoparticles improved the rate of thrombolysis by about 50%. *In vitro* thrombolysis tests using the coated nanoparticles also showed nearly complete removal of thrombus in the microchannel in about 180 seconds. The study has limitations, including the use of a microfluidic channel model, which may not represent the *in vivo* conditions accurately. Also, the study only evaluated the efficiency of the coated nanoparticles compared to pure urokinase solution, and the efficiency of the coated nanoparticles compared to other thrombolytic agents was not evaluated. Future studies should focus on evaluating the efficiency of the coated nanoparticles compared to other thrombolytic agents and *in vivo* testing to evaluate the feasibility of using the nanoparticles for thrombolysis. In addition, as shown in Fig. 7(D), the urokinase-administrated thrombolysis with rotating MNPs under a rotational magnetic field demonstrated that the lysis efficiency is significantly increased by nearly two-fold compared with the same dose of urokinase alone [55]. The experiment also shows that the addition of magnetic nanoparticles can improve the thrombolysis effect when using low dose urokinase. The study has constructed a theoretical model based on convective diffusion to describe the thrombolysis mechanism, and the thrombus lysis speed was determined by the change of the thrombus dissolution length with time in the microfluidic channel. However, the study is limited to *in vitro* experiments, and it remains to be seen how well the approach will translate to *in vivo* conditions. Moreover, the study did not investigate potential toxic effects of the magnetic nanoparticles, and more work is needed to ensure the safety and biocompatibility of the nanoparticles. Meanwhile, urokinase-conjugated water-soluble MNPs have been developed to enhance the efficiency of thrombolysis with manipulation under alternating magnetic fields, and the thrombolytic efficiency is increased nearly four times compared to pure urokinase [122].

Moreover, magnetic nanoparticle micro-swarm could be navigated in a micro-channel towards the target blood clot and deformed under the modulated oscillating magnetic field to realize optimal thrombolysis with tPA under ultrasound imaging [123]. In this study, the microswarm is generated in blood using an oscillating magnetic field and can be navigated with locomotion along both the long and short axis. The aspect ratio of the microswarm can be reversibly tuned by modulating the input field, enabling it to adapt to different confined environments. Under ultrasound imaging, the microswarm is navigated towards a blood clot and deformed to obtain optimal lysis. The study reports a 2.5-fold increase in lysis rate (reaching −0.1725 ± 0.0612 mm^3^/min) in the 37 °C blood environment under the influence of the microswarm-induced fluid convection and tPA compared to that without the microswarm (−0.0681 ± 0.0263 mm^3^/min). In this way, similar results were obtained in a later study with a reconfigurable magnetic micro-swarm to enhance the mass transfer and shear stress near the clot-fluid interface to accelerate tPA-mediated thrombolysis by 3.13-fold compared to tPA only case [124]. The aspect ratio of the microswarm can also be tuned to adapt to different clot regions, and simulations show that the microswarm induces 3D fluid convection that enhances mass transfer and shear stress near the clot-fluid interface. However, the study only focuses on *in vitro* experiments, and the *in vivo* performance of the microswarm has not been investigated. In addition, the long-term biocompatibility and toxicity of the microswarm need to be further evaluated before its clinical use. Moreover, the proposed system requires precise control and localization under ultrasound imaging, and the practicality of this system in clinical settings still needs to be further investigated. In a further study, a magnetite nanoparticle swarm has been developed to induce a hydrodynamic effect to capture tPA in emergent vortices under the dynamic magnetic field, and the dynamic spinning motion of nanoparticles could assist the clot removal by mechanical force to lose fibrin networks that help tPA drug to dissolve the clot completely [125]. The performance of the swarm is tested *in vitro*, and the results show that it can completely remove a 3-mm-diameter and 9-mm-length clot in two hours, which is about three times faster than the clinical procedure applying t-PA alone. The rotation frequency of the swarm significantly increases the removal rate up to a certain point, but sharply drops after reaching the step-out frequency. The concentration of magnetic suspension also affects the time to complete the clot removal, with higher concentrations leading to faster removal times up to a certain point. One limitation of the study is that the experiments were conducted *in vitro*, and further studies are needed to evaluate the feasibility and safety of this approach *in vivo*.

*In vitro* thrombolysis in a dynamic magnetic field offers a promising approach to investigate and develop novel clot-dissolving therapies, but several gaps and future directions need to be addressed for optimization and advancement. Crucial steps include translating *in vitro* findings to *in vivo* models for evaluating safety, efficacy, and real-world applicability, as well as optimizing magnetic field parameters such as strength, frequency, and duration in various *in vitro* settings. Refining magnetic coil and device designs will also contribute to this process. Developing effective targeting strategies to concentrate thrombolytic agents at the clot site in *in vitro* models, along with investigating various magnetic particles and drug carriers, will improve efficacy and safety. Creating standardized *in vitro* models that mimic physiological conditions will enhance comparability and reproducibility across studies. Research should also focus on understanding the underlying mechanisms of clot breakdown in *in vitro* settings, including mechanical forces, thermal effects, and chemical interactions, and developing new thrombolytic agents, magnetic particles, and materials. Addressing these gaps and future directions will help advance *in vitro* thrombolysis in a dynamic magnetic field, potentially leading to improved understanding and optimization of this approach for treating blood clots.

### D. IN-VIVO THROMBOLYSIS IN A DYNAMIC MAGNETICFIELD

**FIGURE 8.**
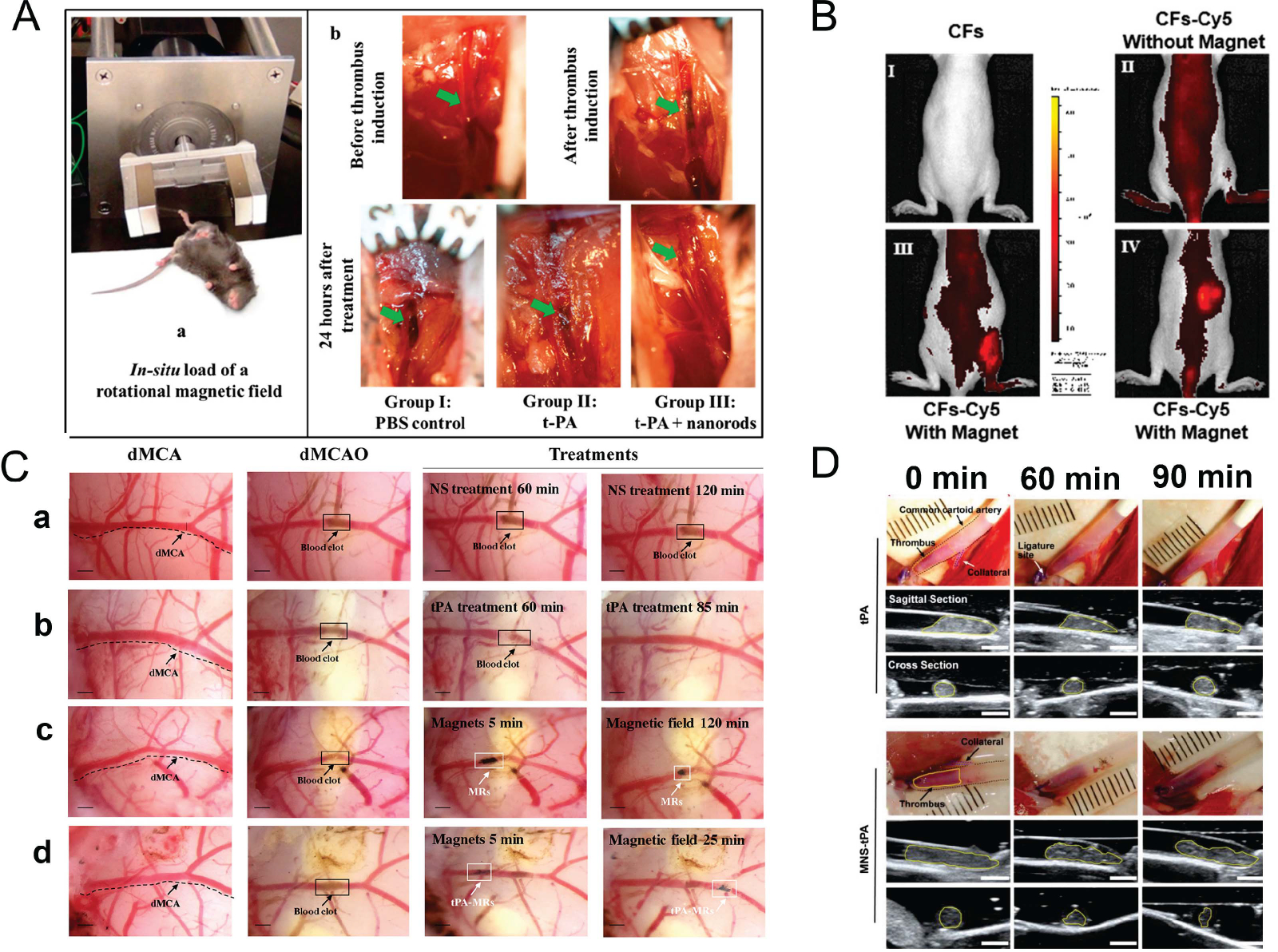
*In-vivo* thrombolysis with magnetic nanoparticles in a dynamic magnetic field. (A) Active nanomotors were used to enhance thrombolysis in a mice embolism model. (a) The experimental setup involved injecting different treatments into the right femoral vessels of C57/BL6 mice and inducing a rotating magnetic field (20 Hz, 40 mT) on the infected hindlimb for 45 minutes. (b) After 24 hours, the mice were anesthetized to check for previously formed thrombi in the femoral vessel. Three groups of mice were used, where Group I was treated with PBS, Group II was injected with t-PA solution, and Group III was treated with both t-PA and nickel rods. The results showed that thrombus remained in all mice of Group I, while Group II mice had a little residual thrombus. However, Group III mice, treated with both t-PA and Ni nanorods and subjected to the rotating magnetic field, had no residual thrombus left. Reproduced from [126], Copyright 2014, with the permission of IEEE. (B) *In vivo* fluorescence images of rats receiving different treatments are shown. Groups III and IV exhibited higher fluorescence intensity at the femoral vein site compared to groups I and II. This suggests that the system was successfully enriched at a specific site with the assistance of a magnet. Reproduced from [127], Copyright 2021, with the permission of IEEE. (C) Representative images of thrombolysis in dMCAO mice under different treatments. (a) As expected, no thrombolysis was observed in the control group treated with normal saline (NS) within 120 minutes. (b) When tPA was administered at a dose of 10 mg/kg, the blood clot was partially lysed and small arteries were reoccluded, resulting in incomplete recanalization within 85 minutes. (c) In contrast, MRs were able to rapidly target the blood clot with the guidance of magnets, but they could not achieve complete recanalization within 120 minutes. (d) However, when tPA-MRs (1 mg/kg; equivalent to 0.13 mg/kg tPA) were administered after dMCAO and under the influence of a rotational magnetic field (RMF, 20 Hz, 40 mT), the blood flow was restored in just 25 minutes, with no occlusion observed in the distal bifurcation. Reproduced from [128], Copyright 2018, with the permission of IEEE. (D) The thrombus images were captured and the changes in the thrombus were measured using ultrasound imaging every 30 minutes for a total of 90 minutes for various treatments. When the carotid artery was obstructed by a thrombus, the injected tPA solution flowed out through the collateral. Microscopic examination did not show any significant change in the thrombus after native tPA solution treatment. However, after 30 minutes of treatment, the signal of the thrombus area in the sagittal ultrasound image weakened, indicating gradual thrombolysis over time. After 90 minutes of treatment, the length of the thrombus decreased but did not dissolve completely, as shown by the filled thrombus signals in the vascular system from the cross-sectional ultrasound image. Combination treatment involved injecting an equivalent amount of tPA solution and magnetic nanoparticles, followed by guided diffusion to the thrombus through rotating locomotion. The thrombus length decreased gradually over time. Notably, the sagittal and cross-sectional ultrasound images revealed an opening at the embolic site at 60 minutes, suggesting recanalization of the blocked blood vessel. The recanalization area further increased at 90 minutes, and a clear passage was formed, as shown in the sagittal ultrasound image. Reproduced from [129], Copyright 2021, with the permission of IEEE.

The feasibility of magnetic nanoparticle-mediated thrombolysis in a dynamic magnetic field was also demonstrated in many *in-vivo* experiments. The rotating magnetic fields can move and rotate the magnetic nanoparticle at high speed and improve drug delivery and penetration efficiency. For instance, as shown in Fig. 8(A), the rotating magnetic nanomotors were developed to enhance the mass transport of tPA drugs near the blood clot surface in a rat embolic model under a rotating magnetic field activation [126]. *In vitro* experiments also show that the thrombolysis speed of low-concentration t-PA can be enhanced up to 2-fold when combined with magnetically activated nanomotors. The study also presents a theoretical model based on convection-enhanced transport due to rotating magnetic nanomotors that predicts the experimental results reasonably well. However, the long-term effects of the nanomotors on the body and their potential toxicity should also be investigated. Moreover, as shown in Fig. 8(B), the dual-response metal-organic-framework (MOF) carbon nanomaterials with the ability to release urokinase to target deep vein thrombosis have been developed to achieve the targeted thrombolysis under the dual-excitation of near-infrared and alternating magnetic fields [127]. The uPA-loaded MOF-derived carbon nanomaterials in this study release urokinase under the action of NIR-mediated photothermy to achieve superficial thrombolysis. When an AMF is applied, the nanomaterials can also heat the thrombosis in the deep tissue area, which leads to enhanced thrombolysis efficiency. The efficiency of the dual-response system was quantitatively evaluated and was found to be nearly 6 times higher than NIR alone. The MOF-derived carbon nanomaterials also demonstrated reliable magnetic targeting ability and angiogenic performance.

In a recent study, as shown in Fig. 8(C), the tPA-coated porous magnetic iron oxide micro-rods have been developed for the targeted cerebral blood clot *in vivo* treatment under an external rotating magnetic field, and the results demonstrated that the rotation of micro-rods could significantly improve both the mass transport of drug and mechanical disruption of fibrin network [128]. The tPA-MRs were able to target the cerebral blood clot *in vivo* under the guidance of an external magnet and when applied with an external rotating magnetic field, improved the mass transport of the tPA-clot reaction and mechanically disrupted the clot network, thus increasing clot interaction and penetration of tPA. The concentration of tPA-MRs in major tissues, including liver, spleen, kidney, lung, and brain, was measured after administration over a period of time until the elimination phase. The maximum concentration in these tissues was within 1 hour after MR injection, with MRs dramatically decreased in the spleen, liver, and kidney 24 hours after administration and barely detected in these organs 12 weeks later. However, further investigation is required to understand the long-term effects of MRs in major tissues and their potential impact on organ function. Moreover, as shown in Fig. 8(D), the C-shaped magnetic actuation system with laser location and ultrasound imaging navigation was developed to guide the tPA and MNPs to the clot site for increased thrombolysis efficacy [129]. The efficacy of the strategy was tested *in vitro* and *in vivo*, and the thrombus length and area were measured from sagittal and cross-sectional ultrasound images. After MNS-tPA treatment for 90 min, the average thrombolysis length was 1.33 ± 0.14 mm (n = 3), and the average sagittal thrombus area decreased to 46.5% ± 3.5% with the MNS-tPA treatment. The MNS-tPA treatment attained the first-rank thrombolysis efficacy with a smaller thrombus area (44.4% ± 0.9%), whereas the area was 91.7% ± 2.1% for the native tPA treatment. The use of MNSs improves the delivery efficiency of tPA and establishes a life channel to achieve time-critical recanalization. However, the study has some limitations such as the small sample size and the lack of long-term safety data. Further studies with larger sample sizes and longer follow-up periods are needed to assess the safety and efficacy of the strategy.

In short, as summarized in Table 3, many studies have demonstrated that MNPs conjugated with approved thrombolytic drugs were helpful for fast, local, and low-risk thrombus therapy. However, more animal studies and clinical trials need to be explored to confirm the safety and efficacy of magnetic nanoparticle-facilitated thrombolytic therapy.

### E. ULTRASOUND AND MAGNETIC DUAL-MODE THROMBOLYSIS

The ultrasound and magnetic dual-mode thrombolysis technique presents multiple benefits for treating blood clots, as it combines ultrasound waves and magnetic targeting to improve the efficiency and safety of thrombolytic therapy [130]. This innovative approach enhances clot breakdown by employing ultrasound waves to generate mechanical forces that break down clots more effectively than chemical thrombolytic agents alone [131], while magnetic targeting concentrates the agent at the clot site [132], [133]. This dual-mode treatment enables the use of lower thrombolytic agent dosages, reducing the risk of bleeding complications [52]. Furthermore, the therapy accelerates clot dissolution, leading to faster treatment, and its non-invasive or minimally invasive nature reduces the risk of infection and other complications. The combination of ultrasound and magnetic targeting minimizes damage to surrounding healthy tissue and avoids systemic effects of thrombolytic agents. Moreover, real-time monitoring via ultrasound imaging allows for adjustments in treatment parameters and early intervention if necessary, making this approach a promising alternative in the treatment of blood clots and potentially leading to better patient outcomes [134], [135].

Many recent studies were focused on developing ultrasound and magnetic dual-mode thrombolysis with magnetic microcarriers, microspheres, and microbubbles. For example, the tPA-encapsulated magnetic microcarriers have been developed to significantly enhance the release of the drug on clot sites under magnetic guidance and ultrasound excitation [136]. The study investigated the encapsulation efficiency, loading, and release of tPA after varying the molecular weight of polymer, carrier size, tPA solution composition, and the use of ultrasound to enhance release. The results showed that the encapsulation efficiency and loading of tPA were dependent on the exact formulation, with tPA concentrations ranging from 3.3–9.4 wt% and magnetite concentrations ranging from 12–17 wt%. The ultrasound waves can cause microbubble oscillations or microstreaming, which mechanically disrupts the microcarriers and enhances the release of tPA. The release of tPA was complete within 20 min after reconstitution, and ultrasound enhanced release rates in larger carriers. The study estimated that 5 mg of carrier concentrated magnetically within about 11 ml of blood volume near the clot should achieve lytic concentrations. In another study, the tPA-loaded magnetic microspheres were used to accelerate the thrombolysis process under magnetic field and ultrasound waves dual-mode excitation [137]. The combination of tPA, magnetic spheres, and a magnetic field showed an improvement in lysis efficiency by 1.7 and 2.7-fold for red and white clots, respectively, under static, no-flow conditions. In dynamic lysis studies, the addition of ultrasound and magnetically-guided spheres to tPA dosages resulted in a 2-fold increase in lysis and a 7-fold reduction in recanalization time. The results showed that the use of magnetically-guided, non-medicated magnetic spheres significantly enhances *in vitro* static and dynamic lysis of red and white blood clots. Besides, a recent study has demonstrated that magnetic microbubbles (MMBs) could release their particle cargo under stable oscillation at resonant frequencies in volume and surface mode. The results showed that the controlled release of MNPs from MMBs could deliver the targeted drug into tissues and improve drug delivery efficiency [138]. MMBs were shown to release doxorubicin-containing poly(lacticco-glycolic acid) particles, and the migration of released nanoparticles from MMBs can be described with a force balance model. The authors demonstrated that this technology can deliver nanoparticles across physiological barriers both *in vitro* and *in vivo*, resulting in an 18-fold and 5-fold increase in nanoparticle delivery to the heart tissue of zebrafish and tumor tissue of mice, respectively. However, it should be noted that the *in vivo* studies were conducted on animal models, and further studies are needed to determine the safety and efficacy of this technology in humans. Additionally, the authors did not report on the clearance of the MMBs and released nanoparticles from the body, which is an important consideration for clinical translation.

Moreover, the feasibility and efficiency of magnetically targeted microbubble-mediated sonothrombolysis for the treatment of blood clots were demonstrated both *in vitro* and *in vivo* with external ultrasound excitation and magnetic targeting methods [52]. The results showed that the US + MMB and US + MMB + r-tPA groups had significantly higher recanalization rate, average blood flow velocity, and hindlimb perfusion than the control and US + MB groups in both red and white thromboembolic models, and the levels of skeletal muscle injury markers were also significantly lower in the US + MMB and US + MMB + r-tPA groups. However, the thrombolytic effects of red thrombi performed better than those of white thrombi in the US + MMB + r-tPA group. Moreover, a recent study showed that the combination of MMBs with tPA under external ultrasound (f = 0.5 MHz, PNP = 0.63 MPa) and magnetic targeting (B = 0.08–0.38 T) could achieve a triple average increase in lysis rate compared to the control group without magnetic targeting [139]. The clot lysis rates were measured by optically monitoring the change in clot diameter in response to the treatment of tissue plasminogen activator, magnetic microbubbles, and ultrasound. A linear correlation was observed between lysis rate and total energy of acoustic emissions. A further study by our group recently demonstrated that intravascular ultrasound thrombolysis with MMBs under a rotational magnetic field could significantly increase the *in vitro* lysis of blood clots [132], [140]. The study investigates the effect of a rotational magnetic field on trapping and vibrating the MMBs at the target clot region, followed by activation by an intravascular forward-looking ultrasound transducer. The role of ultrasounds and RMF was to stimulate the MMBs and enhance their activity at the target site, leading to an increase in clot lysis rates. The study evaluated the impact of different parameters such as blood flow conditions, vessel occlusion conditions, clot ages, ultrasound parameters, and rotational magnetic field parameters on thrombolysis rate. However, the study is limited to *in vitro* experiments, and further research is required to validate the effectiveness of the proposed method *in vivo*. Additionally, the study only investigates the effect of the rotational magnetic field on trapping and vibrating the MMBs, but the impact of this on the thrombolysis rate is not clear. Further research is required to understand the underlying mechanisms of the proposed method and optimize the parameters to achieve maximum efficacy. In addition, the later study from our group demonstrated that the combination of MMBs and nanodroplets could significantly enhance *in vitro* lysis of retracted blood clots under the ultrasound and rotational magnetic field dual-excitation [130]. The study investigated the influence of different treatment methods on thrombolysis rate, including the effects of tPA, MMBs/NDs concentration ratio, and sonication and magnetic field factors. The results showed that combining MMBs and NDs with tPA significantly enhanced *in vitro* lysis of both unretracted and retracted clots, with an unprecedented lysis rate of 85 ± 8.3% for unretracted clots and 57 ± 6.5% for retracted clots in a flow model with 30 min treatment. The magneto-sonothrombolysis rate is higher than that of US only intravascular sonothrombolysis we reported so far [141], [142], [143], [144]. However, it should be noted that the study was conducted *in vitro* and further studies are needed to assess the feasibility and safety of this approach *in vivo*. Additionally, the study did not investigate the potential effects of combining MMBs and NDs with other thrombolytic agents or the long-term effects of this approach.

Magnetic and ultrasound dual-mode thrombolysis shows potential for treating blood clots, but several gaps and future directions warrant further exploration. Researchers need to optimize treatment parameters, such as ultrasound frequency, intensity, and duration, along with magnetic field strength and targeting strategies. Additionally, long-term safety and efficacy assessments, as well as clinical trials, are necessary to validate this approach in comparison to conventional therapies. Moreover, exploring the synergistic effects of dual-mode thrombolysis with other therapies, such as antiplatelet or anticoagulant drugs, may improve treatment efficacy and reduce clot recurrence risk. Developing novel thrombolytic agents, magnetic particles, and ultrasound-responsive materials can further enhance the effectiveness and safety of this technique. Lastly, understanding the underlying mechanisms of clot breakdown, including mechanical forces, thermal effects, and chemical interactions, will advance the development of magnetic and ultrasound dual-mode thrombolysis, potentially leading to improved treatment options and outcomes for patients with blood clots.

*In-vivo* thrombolysis using a dynamic magnetic field is a promising approach for treating blood clots, but there are still gaps and future directions that need to be addressed to optimize and advance this technique. Researchers should focus on determining the optimal magnetic field parameters, including strength, frequency, and duration, as well as developing ideal designs for magnetic coils and devices to ensure effective and safe thrombolysis. Additionally, enhancing targeting strategies to concentrate thrombolytic agents at the clot site will further improve the efficacy and safety of *in-vivo* thrombolysis in a dynamic magnetic field.

In brief, as summarized in Table 4, magneto-sonothrombolysis is a promising approach for the minimal-invasive treatment of blood clots. However, the efficacy and safety of this technique must be demonstrated *in-vivo* in the related animal model and approved in clinical trials for future studies.

## IV. CONCLUSION

In conclusion, many recent studies have been explored to improve thrombolysis efficacy and safety using MNPs with encapsulated thrombolytic drugs. The magnetic targeting delivery of a specific dosage of thrombolytic drugs can effectively shorten the thrombolysis treatment period and prevent hemorrhagic complications compared with conventional treatment with free drugs, which have great potential for clinical applications. For future directions, the stability, biocompatibility, and safety of thrombolytic drug loaded MNPs need to be improved for clinical application. Moreover, ultrasound could be used as an adjuvant to provide feedback for the motion control of MNPs. The promising outcomes from recent studies reveal that fast and effective fibrinolysis on a clot could be possible by applying a combination of MNPs, thrombolytic drugs, ultrasound, and an external magnetic field.

## Figures and Tables

**TABLE 1:** *In-Vitro* Thrombolysis With Magnetic Nanoparticle Under Static Magnetic Field

Particle name	Particle Concentration	Loaded drug type	Main formulation	Static magnetic field	Saturated magnetization	Ref./Year
Activated magnetic modifier	12.1 μg/mL magnetic urokinase	Urokinase 13.8 × 10^4^ IU/mg	Magnetite, α,ω-dicarboxymethylpoly, N-hydroxy-succinimide	Permanent magnet 250 Oe	N/A	[66] 1987
Activated magnetic modifier 30–60 nm	1100 IU/mL magnetic urokinase	Urokinase 1.58 × 10^5^ IU/mg	Magnetite, Polyethylene-glycol derivative	Permanent magnet 250 Oe	N/A	[67] 1988
Magnetic Microspheres 8–53 μm	5 mg/mL magnetic carriers	tPA 5.3–19.6 μg/mL	Magnetite, PLGA, PLA-PEG	Permanent Magnet lOkOe	6.9–8.7 emu/g	[68] 2007
Magnetic nanoparticle	5 mg CMD-MNP	rtPA 0.25 mg	Magnetic nanoparticles Carboxymethyl dextran	Permanent Magnet	N/A	[69] 2011
Magnetic Nanoparticle 22–53 nm	62 μg MNP	tPA Streptokinase 100 IU/mL	SiO_2_-magnetic nanoparticle, Triethoxysilane, Dimethylformamide, Glutaraldehyde	Magnetometer 0–10 kOe	50 emu/g	[71] 2015
Magnetic Nanoparticle 65–170 nm	25 –75 μg Fe/mL	tPA 0.5 mg/mL	SPIONs Carboxymethyl dextran	Susceptometer 0–4000 kA/m	391 ± 3.5 kA/m	[72] 2017
Magnetite sol-gel matrix <500 nm	100 μL thrombolytic sol	Streptokinase 97 kU/mL	iron (II) chloride tetrahydrate, iron (III) chloride hexahydrate, ammonia.	Permanent Magnet 8000 Oe	61 emu/g	[73] 2017
Magnetite nanocontainer 130–250 nm	250 μg/mL MNCs	tPA 600 U/mL	Cyclohexane, n-hexanol, iron (II) chloride tetrahydrate, iron (III) chloride hexahydrate.	Permanent Magnet 15000 Oe	60 emu/g	[74] 2018
Magnetic Composite 400–500 nm	0.05–0.5 mg/mL composite particles	Streptokinase 11.2 kU/mL	SiO_2_ magnetic particle, isobomyl methacrylate, (3-acrylamidopropyl) trimethylammonium chloride	Permanent Magnet 20000 Oe	46 emu/g	[76] 2020
Magnetic Composite 62–166 nm	0.4 mg/mL nanoaggregates	Urokinase 84 kU/mL	Magnetite hydrosol, Anionic polyelectrolytes (heparin, enoxaparin, polyacrylic, polystyrene sulfonate)	Permanent Magnet 8000 Oe	40 emu/g	[77] 2020
Magnetic nanoparticle 148–168 nm	600 IU/mL NK-MNP	Nattokinase, 50–150 IU/mL	Fe_3_O_4_ Carboxymethyl chitosan Sodium Alginate	Permanent Magnet	N/A	[78] 2021
Magnetic nanoparticle 10 nm	1 mg MNP	Streptokinase 1 mg/mL	ZnFe_2_O_4_ nanoparticle, Tetraethyl orthosilicate, Triethoxysilane	Permanent Magnet 3000 Oe	15 emu/g	[79] 2021

**TABLE 2:** *In-Vivo* Thrombolysis With Magnetic Nanoparticles Under a Static Magnetic Field

Model	Particle name/size/dose	Loaded drug type/Dose	Main formulation	Static magnetic field	Saturated magnetization	Ref./Year
Canine carotid arteries model	Magnetic carrier	Streptokinase	Streptokinase Magnetic nanoparticle	Permanent magnet	N/A	[82] 1988
Rat embolic model, iliac artery	Magnetic nanoparticle 250 nm 1 mg/kg	rtPA 1 mg/kg	Nanomag^®^-D (Dextran-coated magnetite)	Permanent magnet (1800–4400 Oe)	N/A	[83] 2007
Primate femoral arteiy model	Magnetic carrier 4.5 μm 10 mg	tPA	PEGylated magnetic nanoparticles	Permanent magnet	40 emu/g	[100] 2008
Rat embolic model	Magnetic nanoparticle 246 nm 2.9 mg/kg	rtPA 0.2 mg/kg	Polyacryhc acid coated- magnetite	Permanent magnet 4900 Oe	61 emu/g	[84] 2009
Rat arteriovenous shunt thrombosis model	Magnetic nanoparticle 116 nm 0.9 mg/kIU	Urokinase 5000–20000IU	Dextran-coated magnetic nanoparticle	Permanent magnet 5000 Oe	24.7 emu/g	[85] 2009
Pig model	Magnetic nanoparticle 140 nm 0.14 mg/ml	tPA 63–71 μg/mg MNPs	Magnetite nanoparticle, triethylene glycol, polyethylene glycol	Permanent magnet 4800 Oe	43 emu/g	[101] 2010
Rat embolism model, iliac artery	Magnetic carrier 14.8 nm 0.2 mg/kg	rtPA 276 μg/mg MNCs	Magnetic nanoparticle, Poly [Aniline-co-N-(l-one-butyric acid) aniline]	Permanent magnet 5000 Oe	52.5 emu/g	[86] 2012
Rat embolic model	Magnetic nanoparticle 139–263 nm 0.2 mg/kg	tPA 0.1 mg/mL	Chitosan-coated magnetic nanoparticle	Permanent magnet 4900 Oe	43.4 emu/g	[87] 2011
Ex-vivo Thrombolysis model	Magnetic nanoparticle 191–200 nm 2 mg/mL	tPA 0.2 mg/mL	Silica-coated magnetic nanoparticle	Permanent magnet 6000 Oe	48.9 emu/g	[88] 2012
Rat abdominal arteries	Magnetic nanoparticle 162–366 nm 20 mg/kg	rtPA 80 μg/mg MNPs	Polyacrylic acid-coated magnetite	Permanent magnet 4900 Oe	N/A	[89] 2014
Rat abdominal aorta	Magnetic nanoparticle 292–395 nm 5 mg/mL	rtPA 1 mg/mL	Poly (lactic-co-glycolic acid), Cychc arginine-glycine-aspartic, chitosan	MRI scanner (3.0 T)	N/A	[90] 2014
Rat embolic model	Magnetic nanoparticle 23–89 nm 0.2 mg/kg	tPA Streptokinase	Silica-coated magnetic nanoparticle	Permanent magnet (5000 Oe)	N/A	[91] 2016
Rat carotid artery/ Rabbit femoral artery	Magnetic nanoparticle 100 nm 0.5–4.2 mg/kg	Urokinase 534 IU/kg for rats and 285 IU/kg for rabbits	Heparin, magnetite nanoparticle	Permanent magnet (4000 Oe)	43 emu/g	[92] 2018
Rat carotid artery	Magnetic nanoparticle 90 nm 1 mg/mL	Nattokinase 1 mg/mL	Silica/polyglutamic acid peptide dendrimer, arginine-glycine-aspartic peptide	Permanent magnet (5000 Oe)	N/A	[94] 2019
Rat tail vein	Magnetic nanosheets 200 nm 100 pg/mL	Heparin 500 μg/mL	PEGylated iron oxide nanoparticle, Polyethyleneimine	Permanent magnet	N/A	[97] 2019
Rat cremaster microvessels	Magnetic nanoparticle 50–250 nm 5 mg/kg	Heparin 100 μg/mL	Dextran-coated magnetic nanoparticle, polyethylene glycol	Permanent magnet (1550 Oe)	N/A	[98] 2019
Rat embolic model	Magneto-liposomes 174– 192 nm 0.2 mg/kg	rtPA 0.2 mg/kg	Citric acid-coated magnetic nanoparticles, DPPC, DSPE-PEG2000	Permanent magnet (5000 Oe)	3.52 emu/g	[93] 2019
Rat tail vein	Magnetic nanoparticle <100 nm 1 mg/mL	Nattokinase 1 mg/mL	Magnetic nanoparticles, polyglutamic acid peptide dendrimer	Permanent magnet	37.5 emu/g	[95] 2020
Rat embolic model	Magnetic nanoparticle 230–321 nm 0.3 U/kg	rtPA 1.5 U/kg	Oleic acid-coated magnetic nanoparticles, Poly (lactic-co-glycolic acid) (PLGA), Avidin, biotin-PEG-maleimide	Permanent magnet (5000 Oe)	<0.3 emu/g	[96] 2020
Rat carotid model	Magnetic nanoparticle 100 nm 5 mg/mL	Thrombin 50–100 U/mL	Carboxy magnetic nanoparticles, NHS, EDC	Permanent magnet	0.27 emu	[99] 2021

**TABLE 3:** *In-Vitro* and *In-Vivo* Thrombolysis With Magnetic Nanoparticles Under a Dynamic Magnetic Field

Study type	Particle name/size/ concentration	Loaded drug type	Main formulation	Saturated magnetization	Dynamic magnetic field	Ref./Year
*In-vitro*	Magnetic nanoparticle 50 nm	N/A	Iron oxide nanopowder	55 emu/g	Oscillating magnetic field (140 Hz, 200–1200 A/m)	[113] 2015
*In-vitro*	Magnetic nanotubes 150 nm 200 μL	tPA 1 mg/kg	Magnetic nanoparticles, albumin, oleic acid, benzyl ether	N/A	Altemating magnetic field (295 kHz, 42 KA/m)	[114] 2015
*In-vitro*	Magnetic nanorods 300 nm 1 mg/mL	tPA 40 μg/mL	Magnetic nanoparticles, 3 -aminopropyltriethoxysilane, dimethyl-formamide	41 emu/g	Rotating magnetic field (20 Hz, 3 mT)	[119] 2016
*In-vitro*	Magnetic liposome 181–443 nm 5 mg/mL	rtPA 0.1 mg/mL	DPPC, DSPE-PEG_2000_, cholesterol, magnetic nanoparticles	4.3 emu/g	Altemating magnetic field	[115] 2017
*In-vitro*	Magnetic micro wheels 1 μm 1.5*10^6^/ μL	tPA 3.6 μg/mL	Streptavidin-coated superparamagnetic nanoparticles	N/A	Rotating magnetic field (100 Hz, 9 mT)	[118] 2017
*In-vitro*	Magnetic nanoparticle 40–200 nm 0.67 mg/mL	Urokinase 0.67 mg/mL	Agar gels-coated magnetic nanoparticle	N/A	Oscillating magnetic field	[121] 2017
*In-vitro*	Magnetic nanoparticle 8–22 nm 0.5 mg/mL	Urokinase 0.5 mg/mL	Citric acid-coated magnetic nanoparticle	N/A	Rotating magnetic field (30 Hz, 3000 A/m)	[55] 2018
*In-vitro*	Magnetic nanoparticle 8–70 nm 1 mg/mL	Urokinase 0.756 mg/mL	Oleic acid-coated magnetic nanoparticle, poly (maleic anhydride-alt-1-octadecylene (PMAO)	N/A	Altemating magnetic field (50–100 Hz, 0.05 T)	[122] 2019
*In-vitro*	Magnetic microrobot 50 μm 5*10^5^ /mL	tPA 1 mg/mL	Magnetic nanoparticle, PEGDE, PEI	<3 emu/g	Altemating magnetic field (375 kHz, 150 Gs)	[116] 2020
*In-vitro*	Magnetic nanoparticle 40 nm 0.5 mg	tPA 10 μg	Magnetic nanoparticle microbeads	N/A	Rotating magnetic field (3 Hz)	[120] 2020
*In-vitro*	Magnetic micro-swarm 400 nm 5 mg/mL	tPA 30 mg/mL	Magnetic nanoparticle	N/A	Oscillating magnetic field (8–20 Hz, 10–12 mT)	[123] 2020
*In-vitro*	Magnetic micro-swarm 43 μm 5 mg/mL	tPA 3 mg/mL	Magnetic nanoparticle	N/A	Oscillating magnetic field (10 Hz, 10–12 mT)	[124] 2021
*In-vitro*	Magnetic nanoparticle 500 nm 0.4 mg/mL	tPA 16–30 μg/mL	Magnetic nanoparticles	>45 emu/g	Rotating magnetic field (0–50 Hz, 30 mT)	[125] 2021
*In-vitro*	Metal-organic carbón nanomaterials 100 nm 0.2 mg/mL	Urokinase 0.2 mg/mL	Iron powder, 1,3,5-trimesic acid, hydrofluoric acid, nitric acid, DBCO PEG cy5	103 emu/g	Altemating magnetic field (318 kHz, 660 Gs)	[127] 2021
*In-vitro*	Magnetic actuators 100–500 nm 20 mg/mL	Urokinase, 0.8 mg/mL	Magnetic nanoparticle, pyrrole, critic acid ligand, ammonium hydroxide	50–155 emu/g	Rotating magnetic field (12–200 Hz, 10–15 mT)	[117] 2021
*In-vivo* (Rat embolic model)	Magnetic nanomotors 0.5–1 μm 7 mg/mL	tPA 10 mg/kg	Polystyrene bead monolayer, Nickel	N/A	Rotating magnetic field (20 Hz, 40–200 mT)	[126] 2014
*In-vivo* (Rat model)	Magnetic microrods 15 nm 5 mg/mL	tPA 0.5 mg/mL	Magnetic micro rods, APTES, dimethyl-formamide	39 emu/g	Rotational magnetic field (20 Hz, 40 mT)	[128] 2018
*In-vivo* (Rabbit carotid artery model)	Magnetic nanoparticle 156 nm 0.5 mg/mL	tPA 10 μg/mL	Magnetic nanoparticles, dopamine, sodium acetate, ethylene glycol	~75 emu/g	Rotating magnetic field (10 mT, 5 Hz)	[129] 2021

**TABLE 4:** Ultrasound and Magnetic Dual-Mode Thrombolysis With Magnetic Microbubbles

Study type	Particle name/size/ concentration	Loaded drug type	Main formulation	Saturated magnetization	Dual excitation type	Ref./Year
*In-vitro*	Magnetic microcarriers 3–53 μm 5 mg/mL	tPA 400 μL	Methoxy polyethylene glycol, stannous octoate, anhydrous toluene, dichloromethane, ether, oleic acid-coated magnetite	6.7–9.9 emu/g	Ultrasound (20 kHz, 50 W), Permanent magnet	[136] 2008
*In-vitro*	Magnetic microspheres ~20 μm 5 mg/mL	tPA 5 μg/mL	Hydrophobic oleic acid-coated magnetite, PLA-PEG	2.8 emu/g	Ultrasound (20 kHz, 38–58 kPa), Permanent magnet (0.4 T)	[137] 2008
*In-vivo* (Mouse tumor model, zebrafish model)	Magnetic microbubbles 450 nm-200 μm 10 mg/mL	Doxorubicin 20 μg/mL	Magnetic nanoparticle, SDS, PLGA	N/A	Ultrasound (10–1000 kHz), Permanent magnet	[138] 2016
*In-vivo* (Rat iliac artery)	Magnetic microbubbles ~2.89 μm 2*10^6^/mL	r-tPA 5 mg/kg	Dipalmitoylphosphatidylcholine, polyethylene glycol-40 stearate, biotin-polyethylene glycol 2000-di-stearoyl phosphatidylethanolamine, propylene glycol, superparamagnetic microbeads	N/A	Ultrasound (2 MHz, MI 1.9), Permanent magnet (5000 Gauss)	[52] 2019
*In-vitro*	Magnetic microbubbles 1–10 μm ~10^7^/mL	tPA 0.75 μg /mL	phospholipid 1, 2-distearoyl-sn-glycero-3-phosphocholine (DSPC), ferrofluid	N/A	Ultrasound (0.5 MHz, 630 kPa), Permanent magnet (0.08–0.38 T)	[139] 2019
*In-vitro*	Magnetic microbubbles ~6 μm ~l0^9^/mL	N/A	Magnetic nanoparticle, SDS	N/A	Ultrasound (620 kHz), Permanent magnet (20–300 Gauss)	[140] 2019
*In-vitro*	Magnetic microbubbles ~6 μm ~10^9^/mL	N/A	Magnetic nanoparticle, SDS	N/A	Ultrasound (620 kHz), Rotational magnetic field (20 Hz, 50 mT)	[132] 2019
*In-vitro*	Magnetic microbubbles, ~6 μm, 10^6^–10^9^/mL Nanodroplet 100–200 nm ~10^8^/mL	tPA 0.75 μg /mL	Magnetic nanoparticle, SDS, DFB microbubbles	N/A	Ultrasound (850 kHz, 2.48 MPa), Rotational magnetic field (40 Hz, 20 mT)	[130] 2021
